# Multiomics-based dissection of citrus flavonoid metabolism using a *Citrus reticulata* × *Poncirus trifoliata* population

**DOI:** 10.1038/s41438-021-00472-8

**Published:** 2021-03-01

**Authors:** Jiaolin Mou, Zhehui Zhang, Haiji Qiu, Yang Lu, Xiang Zhu, Ziquan Fan, Qinghua Zhang, Junli Ye, Alisdair R. Fernie, Yunjiang Cheng, Xiuxin Deng, Weiwei Wen

**Affiliations:** 1 Key Laboratory of Horticultural Plant Biology, College of Horticulture and Forestry Sciences, Huazhong Agricultural University, Wuhan, 430070 China; 2 Thermo Fisher Scientific, Shanghai, 201206 China; 3 National Key Laboratory of Crop Genetic Improvement, Huazhong Agricultural University, Wuhan, 430070 China; 4 Max-Planck-Institute of Molecular Plant Physiology, Am Müehlenberg 1, 14476 Potsdam-Golm, Germany

**Keywords:** Secondary metabolism, Agricultural genetics

## Abstract

Deciphering the genetic basis of plant secondary metabolism will provide useful insights for genetic improvement and enhance our fundamental understanding of plant biological processes. Although citrus plants are among the most important fruit crops worldwide, the genetic basis of secondary metabolism in these plants is largely unknown. Here, we use a high-density linkage map to dissect large-scale flavonoid metabolic traits measured in different tissues (young leaf, old leaf, mature pericarp, and mature pulp) of an F_1_ pseudo-testcross citrus population. We detected 80 flavonoids in this population and identified 138 quantitative trait loci (QTLs) for 57 flavonoids in these four tissues. Based on transcriptional profiling and functional annotation, twenty-one candidate genes were identified, and one gene encoding flavanone 3-hydroxylase (*F3H)* was functionally verified to result in naturally occurring variation in dihydrokaempferol content through genetic variations in its promoter and coding regions. The abundant data resources collected for diverse citrus germplasms here lay the foundation for complete characterization of the citrus flavonoid biosynthetic pathway and will thereby promote efficient utilization of metabolites in citrus quality improvement.

## Introduction

Citrus plants, belonging to the genus *Citrus* L. of the family Rutaceae, are widely grown in tropical and subtropical areas worldwide and are among the most important fruit crops in the world^1,2^. The global citrus production was 152.4 million metric tons in 2018 (http://www.fao.org/faostat/en/#data/QC), ranking first among all fruit crops. Citrus fruits and juices provide us with rich natural products containing high amounts of vitamin C, carotenoids, and flavonoids, thereby conferring significant nutritional and pharmacological benefits^3,4^.

As the largest class of polyphenols, estimated to comprise over 8000 metabolites, flavonoids are widely distributed in the plant kingdom^5^. They usually exist in multitudinous decorated forms, and modification of their common phenol moiety is catalyzed by glycosyltransferase, acyltransferase, hydroxylase, and methyltransferase^6^. These diverse forms enhance the solubility and stability of flavonoids in plants and have a variety of functions. Flavonoids play important roles in several plant biological and physiological processes, such as ultraviolet-B (UV-B, 280–315 nm) protection, which is becoming increasingly critical for obtaining stable yields in deteriorating environments^7,8^. In addition, their pharmaceutical effects on human health as a dietary source of antioxidants have attracted increasing attention^9,10^.

Citrus plants are among the most important dietary sources of flavonoids, which influence their flavor and medicinal value^11,12^. Flavonoids exhibit tissue and species specificity in citrus plants^13^. The major flavonoids in citrus fruits are flavanone-*O*-glycosides, flavone-*O*/*C*-glycosides, and their derivatives^14^. Rich citrus resources provide favorable opportunities for the analysis of natural variation in flavonoids. Studies on specific flavonoids among citrus varieties have been carried out to screen outstanding citrus germplasms and breed citrus plants with optimal levels of these target compounds^15,16^. However, a global view and a systematic understanding of flavonoid diversity in citrus plants and the underlying genetic mechanisms are still lacking.

Reverse genetic approaches have allowed direct determination of the effects of the deficiency, or surplus, of a given protein on the biochemistry of plants^17^. In parallel, top-down approaches, which rely on the screening of a broad range of natural genetic resources for metabolic diversity, have also been taken^18^. In recent years, forward genetic approaches have been successfully applied to dissect plant metabolic traits, and a considerable number of metabolic quantitative trait loci (mQTLs) have been identified in model plants alongside several horticultural plants^19,20^. For example, four QTLs responsible for α-farnesene production in ripe fruit were identified in a segregating ‘Royal Gala’ × ‘Granny Smith’ population, with one major QTL on LG10 colocalizing with the *MdAFS1* (*α-farnesene synthase-1*) gene in apple^21^. Concomitantly, two high-density maps based on two F_2_ populations were developed, and two novel loci, namely, *qgf5.1* and *qgf3.1*, which regulate green-flesh formation resulting from the accumulation of chlorophyll in cucumber, were characterized^22^. In citrus plants, several fruit aroma QTLs and candidate genes in the terpenoid biosynthetic pathway were found using a mandarin linkage population^23^. Recently, based on a high-density integrated genetic map containing 3817 specific lengths amplified fragment (SLAF) sequencing-based molecular markers constructed from a *Citrus* reticulata × *Poncirus* trifoliata F_1_ pseudo-testcross population (used in this study), a total of 17 significant QTLs responsible for carotenoid content in citrus pulp were detected^24^.

Here, we performed ultra-performance liquid chromatography coupled with electrospray ionization and mass spectrometry (UPLC-ESI-MS/MS)-based metabolite profiling in multiple tissues (young leaf, old leaf, mature pericarp, and mature pulp) of 94 lines from the abovementioned *Citrus. reticulata* × *Poncitrus. trifoliata* F_1_ pseudo-testcross population^25^. The same metabolite profiling was also conducted on 14 varieties covering six citrus taxa. QTLs associated with the flavonoid content in these four tissues were detected based on the abovementioned high-density integrated genetic map. By combining transcriptomic analysis, gene functional annotation, and correlation analysis between metabolites and gene expression levels, twenty-one genes were identified, and an important structural gene, *CitF3H*, in the flavonoid pathway was characterized. The function of *CitF3H* in flavonoid biosynthesis has been elucidated in several other plant species; however, its functional verification and naturally occurring functional genetic variation in citrus plants have not yet been reported^26^. This study validates the role of *CitF3H*, responsible for the biosynthesis of dihydrokaempferol; a naturally occurring allelic variation of this gene exerted its effects at the enzyme activity and transcriptional levels in citrus plants. Taken together, the findings of this study lay a foundation for elucidating the citrus flavonoid pathway and provide useful resources to support breeding and engineering efforts for citrus quality improvement and the biosynthesis of beneficial natural products.

## Materials and methods

### Plant materials and growth conditions

The *C. reticulata* (*C. reticulata* Blanco, female parent) × *P. trifoliata* (*P. trifoliata*, male parent) F_1_ population, including 94 siblings, was established in 2003^25^ and used for linkage analysis in this study. In addition, 14 citrus varieties, including *C. grandis* ‘Gaoban’ (GBY), *C. grandis* ‘Siam acidless’ (TAI), *C. aurantium* ‘Daidai’ (DD), *C. sinensis* ‘Washington’ (HSD), *C. sinensis* ‘Valencia’ (XC), *C. reticulata* ‘Mangshanyeju’ (MS), *C. reticulata* ‘Daoxianyeju’ (DX), *C. chuana* ‘Bendizao’ (BDZ), *C. unshiu* ‘Wenzhou’ (WZ), *C. medica* ‘Foshou’ (FS), *C. limon* ‘Eureka’ (YLK), *F. hindsii* ‘QK’ (QK) and *F. crassifolia* (PTJG), and the hybrid citrus variety *C. grandis* × *C. sp*. ‘Sugan’ (SG) were used for gene–metabolome association analysis. The citrus germplasms were collected from the National Citrus Breeding Center of Huazhong Agricultural University, Wuhan, China. Young and old leaves were collected in May and September 2018, respectively (Fig. S1). All fruits were collected at full maturity (at 210 days after flowering; 210 DAF) in 2018 (Fig. S2) and subsequently dissected to obtain two tissues, namely, pericarp (OF) and pulp (OJ), using sterilized scalpels. For each genotype of the F_1_ population, three biological replicates were used for metabolite profiling. Two biological replicates were sampled for metabolite profiling, and one pooled sample was used for transcriptomic analysis in each tissue of the 14 citrus varieties.

### Reagents and standards

HPLC-grade acetonitrile, ethyl acetate, and methanol were purchased from Merck (Darmstadt, Germany). Water was purified using a Milli-Q ULTRA purification system (Millipore, Vimodrone, Italy). Authentic standards of eriocitrin, linarin, hesperidin, naringin, naringenin, methyl hesperidin, didymin, neodiosmin, dihydrokaempferol, and poncirin were purchased from Yuanye S&T Co. (Shanghai, China) (Data S1). Stock solutions of authentic standards were prepared as follows: the compounds were first dissolved in dimethyl sulfoxide and then in methanol.

### Sample preparation and metabolite profiling based on LC-MS

Samples were homogenized to fine powders using liquid nitrogen and then stored at −80 °C until use. One hundred milligrams of freeze-dried powder were weighed and extracted overnight at 4 °C using 1.0 mL of 70% methanol.

The extract was first analyzed by liquid chromatography coupled with Q Exactive Plus mass spectrometry (LC-MS; Thermo Fisher Scientific, California, USA). The mass spectrometer was operated in full-scan data-dependent MS/MS acquisition mode. Characteristic peaks from the liquid chromatography coupled with high-resolution mass spectrometry (LC-HRMS) runs were processed using the commercial software Compound Discoverer (Thermo Fisher Scientific, California, USA). Putative flavonoids were annotated by the mzCloud and mzVault libraries integrated in Compound Discoverer. After identification, the precursor ions and product ions as well as retention times of all the target compounds were exported for subsequent analysis. Selective reaction monitoring (SRM) was performed using triple-quadrupole MS (TSQ Altis; Thermo Fisher Scientific, California, USA). Briefly, high-resolution mass spectra of precursor and product ions as well as the retention times acquired from previous nontargeted metabolite profiling were selected and transferred to construct a transition list for the SRM assay. Then, metabolites of all samples were detected in the SRM scanning mode. The LC conditions were set as follows: solvent system: 0.1% formic acid in water (solution A) and ACN (solution B); gradient program: 0–0.5 min, 98% A; 0.5–15 min, 70% A; 15–28 min, 2% A; 28–28.1 min, 98% A; 28.1–30 min, 98% A; flow rate: 0.4 mL/min.

The relative intensity of each sample was normalized by determining the average of the two adjacent quality controls after deducting the intensity of the blank sample. A data matrix containing the intensity of 80 flavonoids in 108 genotypes from 1041 runs (94 siblings × three samples × four tissue sets of F_1_ population and 14 citrus varieties × two samples × two-three tissue sets) was generated.

### QTL mapping

A SNP-based high-density genetic map of the *C. reticulata* × *P. trifoliata* F_1_ population was previously constructed^24^. Metabolite and expression quantitative trait locus (mQTL and eQTL) detection was carried out using MapQTL software (version 6.0) with the interval mapping (IM) algorithm. The final QTL confidence intervals were assigned as the 1-LOD drop of the peak^27^. In this study, a QTL designated with a LOD value higher than 5 was counted and further analyzed.

### Gene expression profiling using RNAseq and qRT-PCR

Total RNA was extracted using the MAGEN RNA Extraction Kit (Magen Biotechnology, Guangzhou, China) following the manufacturer’s instructions. The library consisted of RNA samples from three different tissues (young leaf, old leaf, and mature pericarp) of 14 citrus varieties. Stranded mRNA-Seq libraries with an insert fragment size of 300 bp were constructed using the KAPA Stranded mRNA-seq Kit (Kapa Biosystems, Cape Town, SA) following the manufacturer’s recommendations and sequenced using the Illumina HiSeq2000 system (paired-end 150-bp reads). Poor-quality reads, adapters, and contaminated reads among the raw reads were trimmed by Trimmomatic software (Version 0.36)^28^ under the default parameters. The clean reads were mapped to the *C. clementina* genome using HISAT (with the -G parameter). The uniquely mapped reads were extracted to estimate the expression levels of protein-coding genes using StringTie. The Ballgown procedure was followed to identify differentially expressed genes (FDR < 0.05)^29^.

Synthesis of the first-strand cDNA and qRT-PCR were conducted as described previously^30^. The gene-specific primers used for qRT-PCR are listed in Table S1.

### Construction of expression plasmids and incubation in yeast

The pESC vector series (Agilent, California, US) was used for the expression of plant genes, and *S. cerevisiae* strain INVSc-1 (Invitrogen) was used as the expression host. Seed cultures were grown for 16–22 h at 30 °C and 210 rpm (New Brunswick C25KC shaker) in 30 mL of selection medium (minus URA) with 2% glucose. After 24 h, 20 μL the old medium was added into 30 mL of fresh medium with 2% glucose, and 24 h later, 1 mL of all substrates (10 μM solutions) was supplied per culture vessel at a final concentration of 0.2 μM along with 2% galactose. Culture broth with cells was harvested after 2 days.

### Extraction of yeast fermentation products and LC-MS detection

Aliquots (500 μL) of culture broth with cells were removed from the culture vessels. When necessary, cells were separated from the culture medium by centrifugation at 14,000 rpm for 1 min. The supernatants were then transferred to another plastic tube, while the cell pellet was suspended in 500 μL of water. All samples were acidified using 25 μL of 6 N HCl and extracted using 550 μL of ethyl acetate. Combined organic fractions were dried in a centrifugal vacuum concentrator. The residues were dissolved in 200 μL of 50% aqueous methanol with 0.1% formic acid and then filtered for LC-MS detection^31^. LC conditions were set as follows: solvent system: 0.05% acetic acid in water (solution A) and 0.05% acetic acid in ACN (solution B); gradient program: 0–1 min, 90% A; 1.1–12 min, 5% A; 12.1–14 min, 90% A; flow rate: 0.3 mL/min.

### Transient overexpression in *N. benthamiana*

*N. benthamiana* was used for transformation. The coding sequence of the *CitF3H* cDNA was amplified and cloned into the PH7WG2D vector. The constructs and vector-only controls were transformed by heat shock into *Agrobacterium tumefaciens* strain GV3101. The monoclonal bacteria were selected in LB containing 100 mg/L spectinomycin and incubated until the OD_600_ was 0.6–0.8. Then, the cells were resuspended in buffer solution (2 mg/mL MES and 2 mg/mL MgCl_2·_6H_2_O), supplemented with 100 mg/L AS and 700 μL of 1 mg/mL naringenin. Tobacco leaves were injected after incubation with buffer solution for 1 h at 28 °C. After 3 days, the leaves were extracted with 70% methanol-water for LC-MS analysis.

### GUS staining

Two types of promoters from *P. trifoliata* and the allele from *C. reticulata* were cloned into the Gateway vector PKGWFS7 and then transferred into *Agrobacterium* strain GV3101. Monoclonal bacteria were selected in LB, incubated until the OD_600_ was 0.6–0.8, and then injected into tobacco leaves. Two days later, the leaves were cut into squares around the injection hole and placed in GUS dye solution. The leaves were kept in the dark at 37 °C and shaken for 24 h, and then, 75% ethanol was used to decolorize the leaves. When all the chlorophyll faded completely, blue areas in the leaves were clearly observed.

### Recombinant protein analysis and enzyme assay

*CitF3H* of *C. reticulata* and the *P. trifoliata* alleles were cloned into the prokaryotic expression vector pET-32a and transformed into *E. coli* strain BL21 (DE3). A single colony was picked and added to 10 mL of LB containing 50 mg/L ampicillin and grown at 37 °C for 5 h. Then, the entire 10 mL LB culture was added into fresh LB (1 L) containing 50 mg/L ampicillin and grown until the OD_600_ reached 0.6–0.8. Isopropyl β-d-1-thiogalactopyranoside (IPTG) was added at 0.5 mM, and the strain was grown at 20 °C and 160 rpm for 16 h. The bacteria were harvested by centrifugation at 4500 rpm for 15 min and washed with lysis buffer (300 mM NaCl, 50 mM sodium phosphate buffer, 10 mM imidazole; pH 8.0). The bacterial pellet was suspended in 10 mL of lysis buffer, and the cells were sonicated 100 times for 4 s with an interval of 4 s. The supernatant was collected by centrifugation at 10,000 rpm for 1 h at 4 °C. The recombinant protein was further purified by Ni-NTA Sefinose resin. The purified protein was washed extensively with elution buffer (300 mM NaCl, 50 mM sodium phosphate buffer, 250 mM imidazole; pH 8.0) and used for enzyme assays and kinetics determination.

The in vitro enzyme activity assay for CitF3H was performed in a total volume of 100 μL containing 200 μM naringenin, 10 mM sodium ascorbate, 0.25 mM ferrous sulfate heptahydrate, 10 mM 2-oxo-glutarate, and 35 μg of purified protein in Tris-HCl buffer (80 mM, pH 8.5), and the mixture was incubated at 35 °C. After incubating for 10 min, the reaction product was extracted by ethyl acetate. The extraction solution was dried by an evaporator, and the residue was resuspended in 200 μL of methanol and then analyzed by LC-MS.

### Kinetic characterization of variants of the CitF3H enzyme

To compare the kinetic constants of recombinant CitF3H proteins with *C. reticulata* and *P. trifoliata* alleles for naringenin acceptors, their activities were determined using a 0–1000 μM naringenin gradient with a fixed amount of purified protein (35 μg). All kinetic parameters were calculated using the Michaelis-Menten model (GraphPad Prism 8.0.1). All reactions were performed in three replicates.

## Results

### Variation in flavonoids in multiple tissues of the *C. reticulata x P. trifoliata* F_1_ pseudo-testcross population

**Fig. 1:**
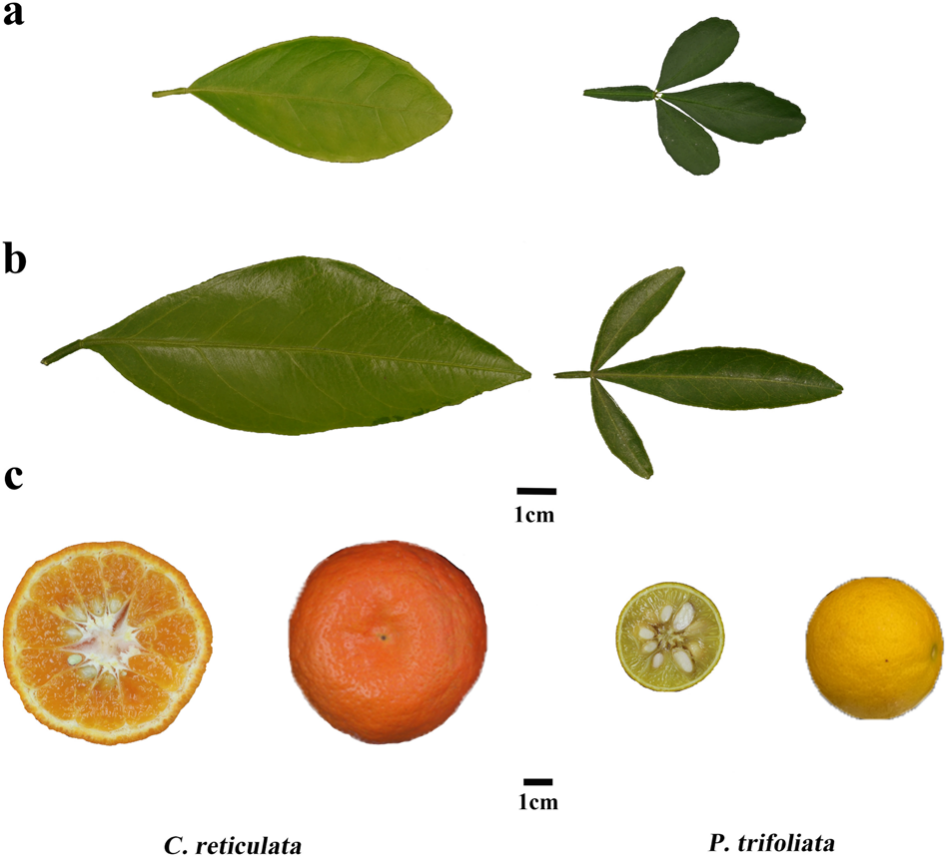
Different tissues of *C. reticulata* and *P. trifoliata*. **a**, **b** and **c** show the young leaf, old leaf, and mature fruit of *C. reticulata* (left) and *P. trifoliata* (right), respectively

Based on liquid chromatography-high-resolution mass spectrometry (LC-HRMS) and liquid chromatography-triple-quadrupole mass spectrometry (LC-TQMS), a total of 80 putative flavonoids were quantified in four citrus tissues spanning the young leaf, old leaf, mature pericarp (210 DAF), and mature pulp (210 DAF) (Fig. 1). Fifty-nine flavonoids were detected in the young leaves, and more than 30 flavonoids were detected in each of the other three tissues (Table S2). These metabolites include flavones, flavonols, flavanones, isoflavones, and anthocyanins, 14 of which were detected in all four tissue types, while 28 were detected in at least three tissue types.

The number of flavonoids with significantly different accumulation between the two types of leaf and mature pulp was greater than that between the two types of leaf and mature pericarp (Table S3). The F_1_ population exhibited great diversity of flavonoids across all four tissue types, especially in the mature pulp. The mean fold changes in flavonoid content among lines were 2.27, 1.56, 14.17, and 71.54 in young leaf, old leaf, mature pericarp, and mature pulp, respectively (Table 1, Table S4). Then, correlation analysis between flavonoids in each tissue was conducted. Flavonoid accumulation patterns varied greatly between leaves and fruits in the F_1_ population. A similar accumulation pattern was observed in mature pericarp and mature pulp, and some of the flavanones showed high correlation, such as cmp550 (hesperidin), cmp1628 (hesperidin isomer(I)), cmp1632 (methyl hesperidin), cmp1239 (naringin isomer(I)) and cmp851 (prunin or isomer(I)). Moreover, a similar metabolic accumulation pattern was also observed between young and old leaves. For instance, the correlation coefficient between cmp829 (naringenin) and cmp856 (prunin or isomer(II)) in young and old leaves reached 0.99 (Fig. S3).

**Table 1:** Range and mean fold changes of metabolic traits measured in the *C. reticulata* x *P. trifoliata* F_1_ Population

Tissue^a^	No. of	Fold change	Fold change
	metabolites^b^	(Mean)^c^	(Range)
YL	59	2.27	1.52–4.89
OL	33	1.56	1.16–5.63
OF	35	14.17	1.69–43.88
OJ	32	71.54	1.49–221.16

^a^YL represents young leaf; OL represents old leaf; OF represents mature pericarp; OJ represents mature pulp

^b^Number of flavonoids detected in this study

^c^Average fold change of all flavonoids in each tissue

### QTL mapping of the flavonoid content

**Fig. 2:**
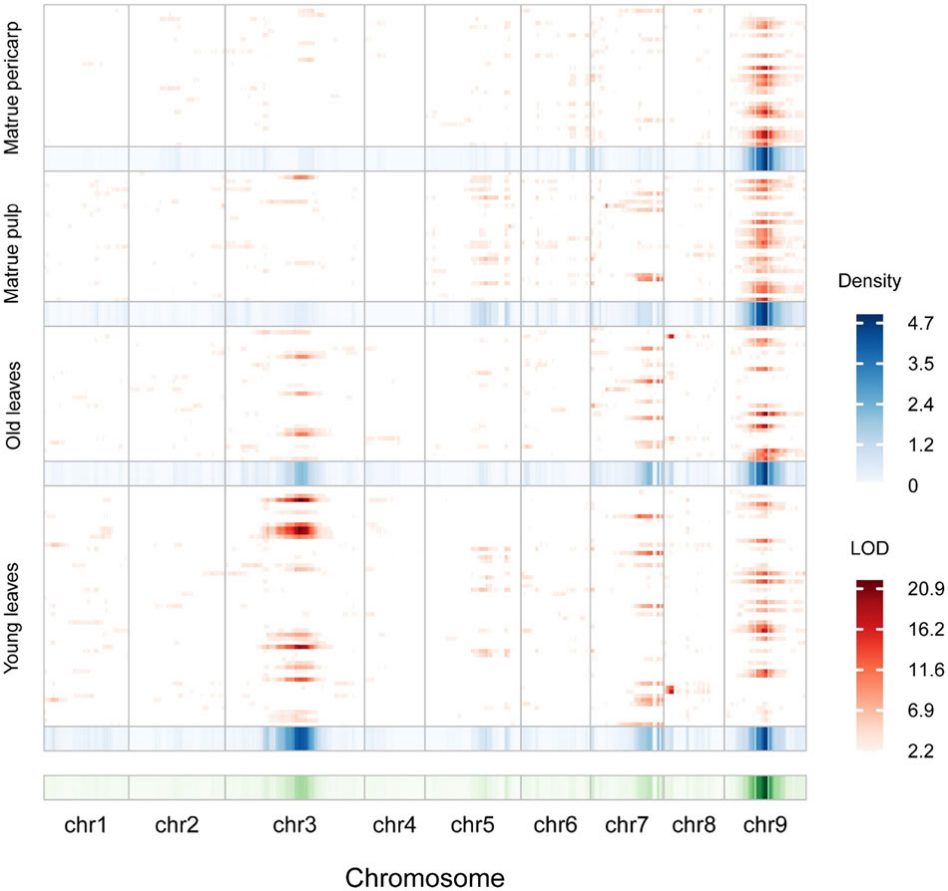
Chromosomal distribution of metabolic QTLs identified in this study. QTL regions (represented by the confidence interval) across the *C. clementina* genome are responsible for metabolite levels from mature pericarp, mature pulp, old leaves and young leaves. The *x* axis indicates genetic positions across the *C. clementina* genome in cM. The heat map under the *x* axis illustrates the density of metabolic QTLs across the genome. The window size is 10 cM. The *y* axis indicates every flavonoid trait detected in the four tissues. The heat map shown in red on the right illustrates LOD values for each trait

QTLs associated with variations in the flavonoid content in the four aforementioned tissues of the F_1_ population were mapped (Fig. 2). A total of 138 QTLs (LOD > 5) affecting the levels of 57 flavonoids were identified. Nineteen QTLs were colocalized in at least three tissues, and two QTLs were present in all four tissues (Table S5).

In the young leaves, 50 QTLs were mapped for 45 metabolic traits. The percentage of phenotypic variation that each QTL could explain ranged from 22.3 to 65.3%, with a mean of 35.0%. In the old leaf, 28 QTLs were mapped for 24 traits. In this case, the phenotypic variation that each QTL could explain ranged from 22.1 to 63.3%, with a mean of 32.3%. Twenty-five QTLs were mapped for 22 traits in mature pericarp, and the phenotypic variation that each QTL could explain ranged from 30.2 to 71.8%, with a mean of 43.4%. By contrast, 35 QTLs were mapped for 26 traits in the mature pulp, with the phenotypic variation explained by each QTL ranging from 26.7 to 63.0%, with a mean of 37.2% (Table 2, Table S6 and Table S7).

**Table 2:** Summary of QTL mapping for metabolic traits measured in the *C. reticulata* x *P. trifoliata* F_1_ population

Tissue^a^	No. of	No. of QTLs	No. of QTLs
	metabolic traits^b^	(Mean)^c^	(Range)
YL	45 (59)	1.11	1–2
OL	24 (33)	1.17	1–2
OF	22 (35)	1.14	1–2
OJ	26 (32)	1.35	1–3

^a^YL represents young leaf; OL represents old leaf; OF represents mature pericarp; OJ represents mature pulp

^b^Number of flavonoids that have at least one QTL identified in this study; the number in parentheses represents all the metabolic traits detected in this tissue

^c^Average number of QTLs identified for each flavonoid

Of the identified QTLs, 13.8% were located on Chr3, 26.1% were located on Chr7, and 46.4% were mapped on Chr9. The remaining 13.7% were distributed randomly across the other chromosomes. Three QTL hotspots (on chromosomes 3, 7, and 9) were identified with an interval size of 8.05 Mb (Ciclev19777128 bp_27830405bp), 3.10 Mb (Ciclev852254bp_3948461bp) and 3.68 Mb (Ciclev17388036 bp_21073008 bp), including 463, 391 and 98 genes, respectively (with reference to the *C. clementina* genome; Table S5, S8 and Fig. 2^32^).

### Candidate gene selection

We further screened candidate genes within the abovementioned hotspots. First, correlations between the expression levels of the 952 genes encoded within the hotspots (Table S9) and the relative metabolite levels (Table S10) were calculated using the transcriptomic and metabolic data collected for the 14 citrus varieties. There were 184, 228, and 278 highly correlated gene-metabolite pairs in the young leaf, old leaf, and mature pericarp, respectively (|R | ≥ 0.8, *p* < 0.01) (Table S11). Among these metabolite-gene correlations, 43 were identified in at least two tissues, and three were found in all three tissues (Table S12). In addition, based on functional annotation, 21 genes putatively associated with the flavonoid pathway were selected. Detailed information, including the corresponding metabolic trait, functional annotation, physical position, tissue type, and approach used for cross-validation, for these 21 candidate genes is provided in Table 3.

**Table 3:** Summary of candidate genes and related information

Gene	Metabolic trait		Functional annotation	Chr	Physical position		Tis	Cross-validation
Ciclev10021069m.g	1532	Eriocitrin isomer(I)	2-oxoglutarate (2OG) and Fe(II)-dependent oxygenase superfamily protein	Chr_3	21579109	21583976	YL	R_G-M_, eQTL
	1232	Kaempferitrin isomer(I)					YL	R_G-M_, eQTL
	1251	Neodiosmin					YL,OL	R_G-M_, eQTL
	1249	Linarin					YL	mQTL, R_G-M_, eQTL
Ciclev10023815m.g	1532	Eriocitrin isomer(I)	2-oxoglutarate (2OG) and Fe(II)-dependent oxygenase superfamily protein	Chr_3	21651769	21652245	YL,OL	R_G-M_
	1232	Kaempferitrin isomer(I)					YL,OL	R_G-M_
	1251	Neodiosmin					YL,OL	R_G-M_
	1249	Linarin					YL,OL	R_G-M_
Ciclev10024496m.g	1632	Methyl hesperidin	S-adenosyl-L-methionine-dependent methyltransferases superfamily protein	Chr_3	26666978	26668075	YL	R_G-M_
	178	3,4-Dihydroxy-6,7,3′,4′-tetramethoxyflavonol					YL	R_G-M_
Ciclev10026967m.g	1027	Hesperetin or isomer(I)	basic helix-loop-helix (bHLH) DNA-binding superfamily protein	Chr_7	3255156	3255959	YL	R_G-M_
	856	Prunin or isomer(II)					OL	mQTL, R_G-M_
	642	Artabotryside A or isomer(I)					OL	R_G-M_
Ciclev10024361m.g	734	Luteolin-3′,7-Diglucoside or isomer(I)	S-adenosyl-L-methionine-dependent methyltransferases superfamily protein	Chr_3	25649848	25653317	YL	R_G-M_
Ciclev10021734m.g	1232	Kaempferitrin isomer(I)	S-adenosyl-L-methionine-dependent methyltransferases superfamily protein	Chr_3	26976583	26978658	YL,OL	R_G-M_
	1249	Linarin					OL	mQTL, R_G-M_
	1532	Eriocitrin isomer(I)					OL	R_G-M_
Ciclev10026557m.g	1232	Kaempferitrin isomer(I)	myb-like transcription factor family protein	Chr_7	2881144	2882746	OL	R_G-M_
	1251	Neodiosmin					OL	R_G-M_
Ciclev10027167m.g	877	Keracyanin or isomer(I)	cytochrome P450, family 722, subfamily A, polypeptide 1	Chr_7	1758740	1762044	OL	R_G-M_
Ciclev10027097m.g	1632	Methyl hesperidin	cytochrome P450, family 722, subfamily A, polypeptide 1	Chr_7	936705	940639	OL	R_G-M_
	178	3,4-Dihydroxy-6,7,3′,4′-tetramethoxyflavonol					OL	R_G-M_
Ciclev10023900m.g	879	Keracyanin or isomer(II)	S-adenosyl-L-methionine-dependent methyltransferases superfamily protein	Chr_3	26724892	26726825	OL	R_G-M_
Ciclev10006413m.g	1251	Neodiosmin	basic helix-loop-helix (bHLH) DNA-binding superfamily protein	Chr_9	20145029	20146263	OL	R_G-M_
Ciclev10027234m.g	829	Naringenin	cytochrome P450, family 81, subfamily D, polypeptide 2	Chr_7	2678933	2679557	OL	R_G-M_
	1620	Naringin					OL	R_G-M_
	856	Prunin or isomer(II)					OL	mQTL, R_G-M_
Ciclev10021728m.g	1232	Kaempferitrin isomer(I)	S-adenosyl-L-methionine-dependent methyltransferases superfamily protein	Chr_3	26847877	26850055	OL	R_G-M_
	1249	Linarin					OL	mQTL, R_G-M_
	1532	Eriocitrin isomer(I)					OL	R_G-M_
Ciclev10025490m.g	660	Methyl hesperidin isomer(I)	UDP-Glycosyltransferase superfamily protein	Chr_7	2356952	2358892	OF	R_G-M_
Ciclev10025311m.g	1249	Linarin	cytochrome P450, family 711, subfamily A, polypeptide 1	Chr_7	1492717	1495961	OF	R_G-M_
	1251	Neodiosmin					OF	R_G-M_
	1232	Kaempferitrin isomer(I)					OF	R_G-M_
	1532	Eriocitrin isomer(I)					OF	R_G-M_
Ciclev10027189m.g	1249	Linarin	O-methyltransferase family protein	Chr_7	1085045	1086475	OF	R_G-M_
	1251	Neodiosmin					OF	R_G-M_
	1232	Kaempferitrin isomer(I)					OF	R_G-M_
	1532	Eriocitrin isomer(I)					OF	R_G-M_
Ciclev10025931m.g	1532	Eriocitrin isomer(I)	flavanone 3-hydroxylase	Chr_7	1207141	1210820	OF	eQTL, R_G-M_
	1249	Linarin					OF	eQTL, R_G-M_
Ciclev10004785m.g	1532	Eriocitrin isomer(I)	cytochrome P450, family 71, subfamily B, polypeptide 34	Chr_9	20764326	20767162	OF	R_G-M_
Ciclev10023355m.g	1249	Linarin	O-methyltransferase family protein	Chr_3	19971303	19972959	OF	R_G-M_
Ciclev10026344m.g	178	3,4-Dihydroxy-6,7,3′,4′-tetramethoxyflavonol	S-adenosyl-L-methionine-dependent methyltransferases superfamily protein	Chr_7	3362860	3364363	OF	R_G-M_
Ciclev10024440m.g	1620	Naringin	myb domain protein 14	Chr_3	23417440	23417807	OF	R_G-M_
	856	Prunin or isomer(II)					OF	R_G-M_
	1027	Hesperetin or isomer(I)					OF	R_G-M_
	879	Keracyanin or isomer(II)					OF	R_G-M_
	829	Naringenin					OF	R_G-M_

^a^YL represents young leaf; OL represents old leaf; OF represents mature pericarp

^b^R_G-M_ represents correlation between gene expression and metabolic level; mQTL represents QTL mapping of flavonoid contents; eQTL represents QTL mapping of relative expression level quantified by qRT-PCR

**Fig. 3:**
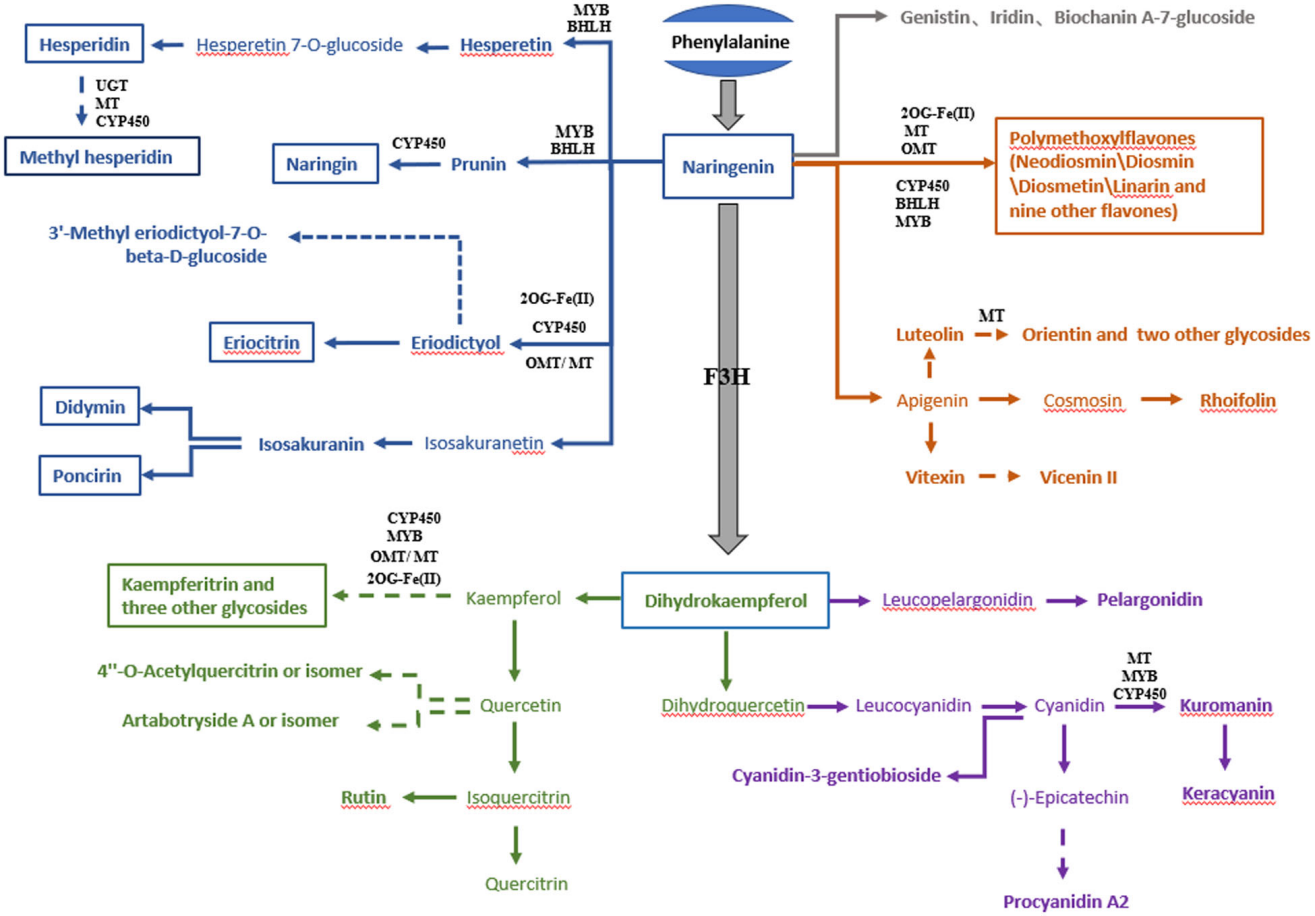
A proposed citrus flavonoid metabolic network. Candidate genes identified in this study are shown in their respective pathways. Metabolites and genes identified in this study are shown in bold. Flavonoids in blue, green, gray, orange and purple are flavanones, flavonols, isoflavones, flavones, and anthocyanins, respectively. The flavonoids in the box were verified by standards. UGT, UDP-glycosyltransferase; MT, S-adenosyl-L-methionine-dependent methyltransferase; *OMT*, O-methyltransferase family protein; *CYP450*, cytochrome P450; *bHLH*, basic helix-loop-helix (bHLH) DNA-binding superfamily protein; *MYB*, myb-like transcription factor family protein; 2OG-Fe(II), 2-oxoglutarate (2OG) and Fe(II)-dependent oxygenase; *F3H*, flavanone 3-hydroxylase

Nine of the candidate genes are located on chromosome 3, including genes encoding an *O*-methyltransferase, a MYB14 domain protein, two 2-oxoglutarate (2OG) and Fe(II)-dependent oxygenase superfamily proteins and five S-adenosyl-L-methionine-dependent methyltransferase superfamily proteins. Ten of the candidate genes are located on chromosome 7, including genes encoding a flavanone 3-hydroxylase (*F3H*), an S-adenosyl-L-methionine-dependent methyltransferase superfamily protein, a UDP-glycosyltransferase superfamily protein, an *O*-methyltransferase family protein, two transcription factors (a MYB-like transcription factor and a basic helix-loop-helix DNA-binding superfamily protein), and four cytochrome P450s. Another two candidate genes located on chromosome 9 are genes encoding a cytochrome P450 (family 71, subfamily B) and a basic helix-loop-helix (bHLH) DNA-binding superfamily protein (Table 3). Among these candidate genes, *Ciclev10025931m.g* encodes a flavanone 3-hydroxylase (*F3H*), which is the key enzyme that converts flavanone (naringenin) to flavonol (dihydrokaempferol) (Fig. 3). Notably, based on the relative expression level profiling conducted using qRT-PCR, eQTLs were identified for 16 candidate genes (Table S13), and the eQTL of *Ciclev10021069m.g* and mQTL of the associated metabolite linarin were also colocalized (Tables S7, S8, and S14). Some candidate genes selected here are likely to function as gene clusters. For example, both *Ciclev10021069m.g* and *Ciclev10023815m.g*, annotated as 2-oxoglutarate (2OG) and Fe(II)-dependent oxygenases, are adjacent to one another.

### A proposed citrus flavonoid metabolic network involving key genes and metabolites

Based on the highlighted candidate genes and metabolites found in this study, a citrus flavonoid metabolic network is proposed (Fig. 3). The network contains five modules of flavonoid synthesis, with three modules starting from naringenin, including flavanones, flavones, and isoflavones and two modules consisting of flavonols and anthocyanins derived from dihydrokaempferol. Within this network, most genes may possess functions in metabolite modification, while most detected flavonoids are downstream products of pathways modified via glycosylation, methylation, oxygen methylation or hydroxylation. Notably, three putative isoflavones (cmp843, cmp1166 and cmp1208) are included within this network, and eight pathway branches (represented by the dotted arrow in Fig. 3) leading to methyl hesperidin and seven other flavonoids, annotated as 3′-methyl eriodictyol-7-*O*-beta-D-glucoside, kaempferitrin, 4”-*O*-acetylquercitrin or an isomer thereof, artabotryside A or an isomer thereof, orientin, vicenin II and procyanidin A2, are reported for the first time here. According to the correlation between gene expression and the level of metabolite accumulation, candidate genes were mapped onto the corresponding pathway branches. For instance, *Ciclev10024496m.g*, which is annotated as an S-adenosyl-L-methionine-dependent methyltransferase and correlated with the levels of cmp178 (3,4-dihydroxy-6,7,3′,4′-tetramethoxyflavonol) and cmp1632 (methyl hesperidin), is very likely associated with the branch of methyl hesperidin.

Notably, some flavonoids in the network have medicinal value and are enriched in *P. trifoliata*, which is utilized as traditional Chinese medicine. For example, here, the linarin content in *P. trifoliata*, which was shown to possess antipyretic, analgesic, and anti-inflammatory activities^33^, was 1672 times higher than that in *C. reticulata*. Moreover, the levels of cmp1234 (rhoifolin) and naringin in *P. trifoliata*, both of which have been regarded as indicators of the efficacy of ECG (*Exocarpium Citri Grandis*, a precious Chinese medicine with a history spanning hundreds of years)^34^, are over 700 times more abundant than those in *C. reticulata* (Table S15).

### Overexpression of *CitF3H* leads to the generation of dihydrokaempferol in yeast and tobacco

**Fig. 4:**
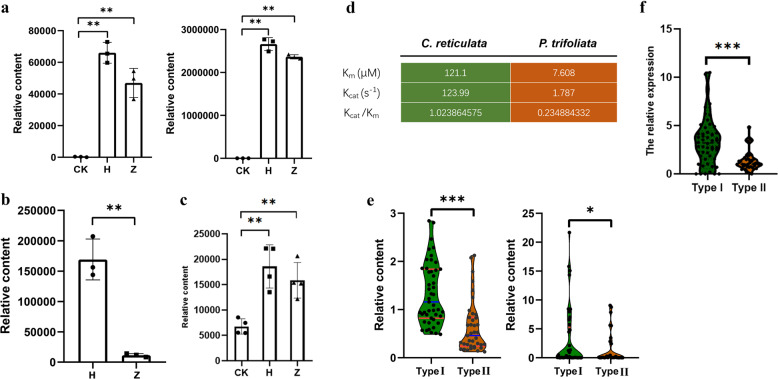
The level of dihydrokaempferol is associated with the genetic variants of *CitF3H* in the coding and promoter regions. **a** Validation of the candidate gene *CitF3H* in yeast. The left panel shows the yeast precipitate, and the right panel shows the supernatant. CK represents the control line, H represents the overexpression line with the *C. reticulata* allele, and Z represents the overexpression line with the *P. trifoliata* allele. **b** The relative content of dihydrokaempferol in the young leaf. **c** The relative content of dihydrokaempferol in the leaves of two transient overexpression lines of *N. benthamiana*. **d** The kinetic parameters of recombinant CitF3H proteins with the *C. reticulata* and *P. trifoliata* alleles, and the data are presented for three replicates in one assay. **e** The relative content of dihydrokaempferol in the type I and type II genotypes. The left panel shows the young leaf, and the right panel shows mature pulp. **f** The relative expression level of *CitF3H* in type I and type II genotypes. All the results are based on the *t*-test of independent samples (*p < 0.05, **p < 0.01 and ***p < 0.001)

Flavanone 3-hydroxylase (*F3H*) is a much-studied upstream structural gene of the flavonoid biosynthetic pathway (Fig. 3 and Fig. S4a). To characterize the function of *CitF3H* (*Ciclev10025931m.g*), yeast lines overexpressing *C. reticulata* and *P. trifoliata* alleles were constructed. Following the provision of the putative substrate naringenin, the supernatant and precipitate of the yeast culture were extracted for metabolite measurement. Dihydrokaempferol was enriched mainly in the supernatant, and the intensity of dihydrokaempferol increased dramatically in the yeast overexpression lines expressing both the *C. reticulata* and *P. trifoliata* alleles. Moreover, the intensity of dihydrokaempferol in the line expressing the *C. reticulata* allele was significantly higher than that with the *P. trifoliata* allele (Fig. 4a and Fig. S4d–i), which is consistent with the content of dihydrokaempferol detected in the young leaves of *C. reticulata* and *P. trifoliata*, respectively (Fig. 4b).

To assess the role of the proteins in vivo, transient overexpression of *CitF3H* in *N. benthamiana* was performed. The content of dihydrokaempferol in lines overexpressing either the *C. reticulata* or *P. trifoliata* allele was significantly higher than that in the negative control. Consistent with the results in yeast, the dihydrokaempferol content in the line expressing the *C. reticulata* allele was higher than that in the line expressing the *P. trifoliata* allele (Fig. 4c). Similar results were observed when the substrate naringenin was coinjected with *Agrobacterium* into tobacco leaves (Fig. S5).

### Identification of functional genetic variants of *CitF3H*

By comparing the coding regions of *CitF3H* of *C. reticulata* and *P. trifoliata*, we found 13 SNPs resulting in five amino acid differences and one indel in *P. trifoliata* (Fig. S6). Recombinant CitF3H proteins of *C. reticulata* and *P. trifoliata* were then expressed and purified for enzymatic assays. The Michaelis-Menten equations of the recombinant CitF3H proteins with the *C. reticulata* and *P. trifoliata* alleles were fitted (Table S16 and Fig. S7). The kinetic parameters of *C. reticulata* were quantified, yielding a *K*_*m*_ (μM) of 121.1 and *K*_cat_ (s ^−1^) of 123.99, while for *P. trifoliata*, the *K*_*m*_ (μM) and *K*_cat_ (s ^−1^) were 7.608 and 1.787, respectively. The recombinant CitF3H protein of the *C. reticulata* allele therefore clearly shows superior catalytic efficiency compared with that of the *P. trifoliata* allele (Fig. 4d).

To further dissect the relationship between the genetic variations in the promoters of *CitF3H* and the content of dihydrokaempferol, the activity of three different types (one is the allele of *C. reticulata*, which is homologous at this locus; the other two are alleles of *P. trifoliata*, which is heterozygous at this locus; Data S2) of promoter sequences was compared through GUS staining. The staining area of the two *P. trifoliata* promoter types was larger than that of *C. reticulata*, and the staining area of the *P. trifoliata* promoter allele A (short deletion) was larger than that of allele B (long deletion; Fig. S8). The promoter activity was consistent with the expression level of *CitF3H* in *C. reticulata and P. trifoliata*. In both the young leaf and mature pulp, the expression level of *CitF3H* in *P. trifoliata* was higher than that in *C. reticulata* (Fig. S9).

Furthermore, a molecular marker designed for discriminating the different promoter sequences was validated in the F_1_ population. The progeny can be grouped into two types based on their genotypes: type I (allele of *C. reticulata* and allele A of *P. trifoliata*) and type II (allele of *C. reticulata* and allele B of *P. trifoliata*) (Fig. S10). The relative intensity of dihydrokaempferol in the type I lines was significantly higher than that in the type II lines (*p* = 0.05, *t*-test; Fig. 4e), which is consistent with the relative expression between the type I and type II lines (*p* = 0.05, *t*-test; Fig. 4f). Hence, genetic variations in both the coding and promoter regions of *CitF3H* are responsible for the variation in dihydrokaempferol content in this population.

## Discussion

Forward genetic studies integrating metabolic profiling with quantitative genetics have been conducted in many plant species, including mainly model plants and crops such as maize, rice, and wheat^35,36^. These studies successfully elucidated the genetic basis underlying metabolic variation. Owing to the long growth cycle and breeding process, it is far more difficult to conduct forward genetic studies in perennial woody plants. However, some important traits, such as fruit flavor and quality, have still been dissected using QTL analysis^37–39^, such as identification of QTLs controlling aroma volatile production in a *Citrus reticulata* population^23^ and mapping of carotenoid content in citrus fruits using a high-density genetic map^24^. As important fruit crops worldwide, citrus plants are rich in specialized metabolites, including flavonoids and carotenoids. Detection and comparison of flavonoids at different developmental stages in different citrus species and tissues and mining of candidate genes related to flavonoid metabolism have been previously performed^40–42^. However, studies based on QTL mapping and identification of candidate genes underlying the flavonoid content in citrus plants are lacking.

By adopting widely targeted metabolomic and transcriptomic analyses and combining results from the segregating population and diverse citrus varieties, this study provided valuable insights allowing the dissection of flavonoid biosynthesis in citrus plants. A number of QTLs overlapped in at least three tissues, indicating that the content of at least some flavonoids may be under common genetic control across different tissue types. However, most QTLs were tissue specific, suggesting distinct genetic and biochemical regulatory bases between tissues, which is consistent with the reported tissue specificity of flavonoids in citrus plants^13,42^. Moreover, the metabolic diversity among different tissues might result from spatially or temporally distinct expression patterns of genes, such as tissue-specific transcript expression and alternative splicing^43^.

Flavanone 3-hydroxylase (F3H) is a key enzyme channeling carbon flow toward the production of 3-hydroxylated flavonoids, including flavonols, proanthocyanidins, and anthocyanidins, and it has been well characterized in a wide range of plant species^44–46^. *Petunia hybrida* tends to accumulate flavonol glycosides and anthocyanins; however, *Citrus paradisi* is known for its accumulation of flavanone diglycosides^47^. Different species display considerably different flavonoid accumulation patterns, which possibly results from the function of F3H. Recently, it was reported that in a sorghum variety lacking endogenous F3H activity, heterologous F3H overexpression led to enhanced accumulation of flavonols and flavan-3-ols as well as considerable changes in the flavonoid profile. However, no such changes in the flavonoid profile occurred in tobacco with endogenous F3H activity, further demonstrating the functional complexity of F3H^48^.

Here, we verified the function of *CitF3H* in citrus plants, demonstrating that it can convert naringenin into dihydrokaempferol. We further revealed that genetic variations in the coding region of *CitF3H* between the two parental lines resulted in differences in its enzyme activity. Moreover, an indel variation in the promoter region of *CitF3H* can affect the content of dihydrokaempferol by varying the transcriptional level of *CitF3H*. Considering the key position of F3H in the flavonoid pathway, these findings regarding natural variations in *CitF3H* could aid in the genetic improvement and synthesis of beneficial flavonoids in citrus plants. It has been indicated that enzymes that enable the formation of flavonoid scaffold structures probably first appeared by recruiting enzymes from primary metabolic pathways. Subsequently, enzymes that belong to superfamilies, such as 2-oxoglutarate-dependent dioxygenase, cytochrome P450, and short-chain dehydrogenase/reductase, modified these structures^49^. Interestingly, most of the 21 candidate genes highlighted here were annotated as downstream modification enzymes, including CYP450s, 2OG-Fe(II)s, methyltransferases, oxymethyltransferases and glycosyltransferases. Enzymes responsible for the final steps of metabolite synthesis generally make greater contributions to the natural variation in metabolite abundance than those earlier in the pathway^5^. Further functional and biochemical investigation of these remaining candidate genes will undoubtably provide insights into the expansion and supplementation of the flavonoid pathway in citrus plants.

Genes in our proposed flavonoid metabolic network were mapped based on the correlation of metabolite accumulation and gene expression level, in which one gene may be associated with multiple metabolites or, conversely, multiple genes may be associated with the same flavonoid. The network contained genes that were adjacent to each other and had the same functional annotation, namely, *Ciclev10023815m.g* and *Ciclev100210*69*m.g*, both of which encode a 2-oxoglutarate (2OG) and Fe(II)-dependent oxygenase. Whether these gene clusters are variable among different citrus species warrants further study. On the other hand, the similarity in the chemical structure of flavonoids presents a massive challenge in the identification of these compounds. In this study, authentic standards and high-resolution *m/z* and MS/MS fragmentation were used for the identification and annotation of flavonoids. That said, further accurate structural determination of additional metabolites would be helpful for elucidation of a more complete network.

Although they exhibit an astringent taste, the *Poncirus trifoliata* [L.] Raf. the fruit has been widely used as a traditional medicine in East Asia owing to the proposed anticancer and anti-inflammatory activities of the metabolites they contain^50^. Some specific flavonoids with important pharmacological value, such as linarin, cmp1234 (annotated as rhoifolin) and naringin, were more enriched in *P. trifoliata* than in *C. reticulata*. Therefore, *P. trifoliata* and the F_1_ population represent useful resources for the improvement of the popular fruit crop *C. reticulata*. The enhancement of compounds with high medicinal value and lacking undesirable flavors, such as bitterness and astringency, could be a direction for future fruit breeding.

In summary, the multiomics analysis based on a citrus genetic population and diverse varieties generated rich data resources and identified a number of QTLs for flavonoid content in multiple tissues, laying a foundation for elucidation of the flavonoid biosynthetic pathway and presents a blueprint that can be followed for unveiling further metabolic pathways in citrus.

## Supplementary information

Supplemental information

Cover letter

Data S1

Data S2

Table S1

Table S2

Table S3

Table S4

Table S5

Table S6

Table S7

Table S8

Table S9

Table S10

Table S11

Table S12

Table S13

Table S14

Table S15

Table S16
