# Supplementary material for: Multiomics-based dissection of citrus flavonoid metabolism using a Citrus reticulata × Poncirus trifoliata population
Source: Hortic Res. 2021 Mar 1;8:56. doi: 10.1038/s41438-021-00472-8 (PMC7917093; doi:10.1038/s41438-021-00472-8)
Supplement: Supplementary file 1 — Supplemental information [file 41438_2021_472_MOESM1_ESM.doc]

**
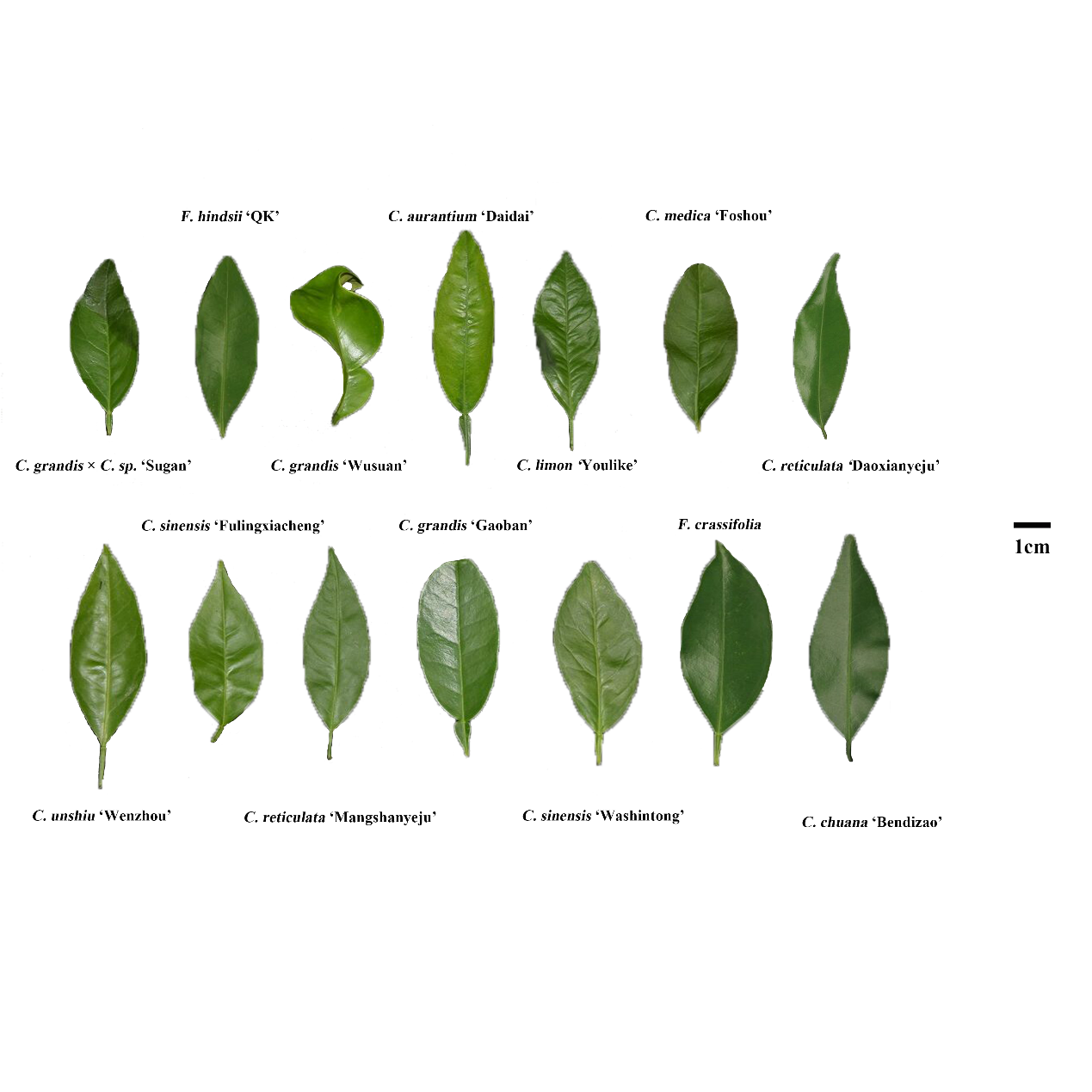
**

**Fig. S1. The Young Leaves of 14 Citrus Varieties in This Study.**


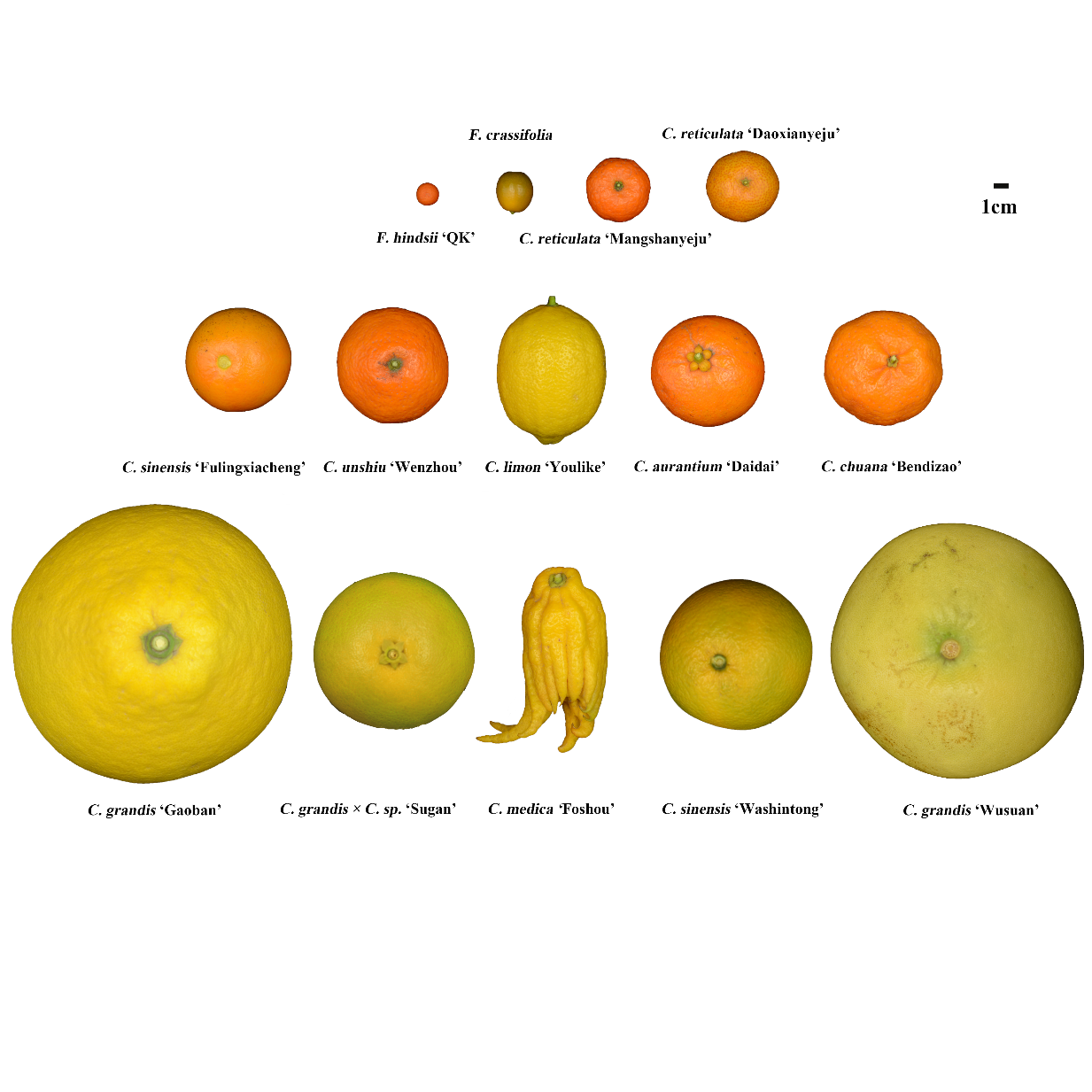
**Fig. S2. The Mature Fruits of 14 Citrus Varieties in This Study.**

**
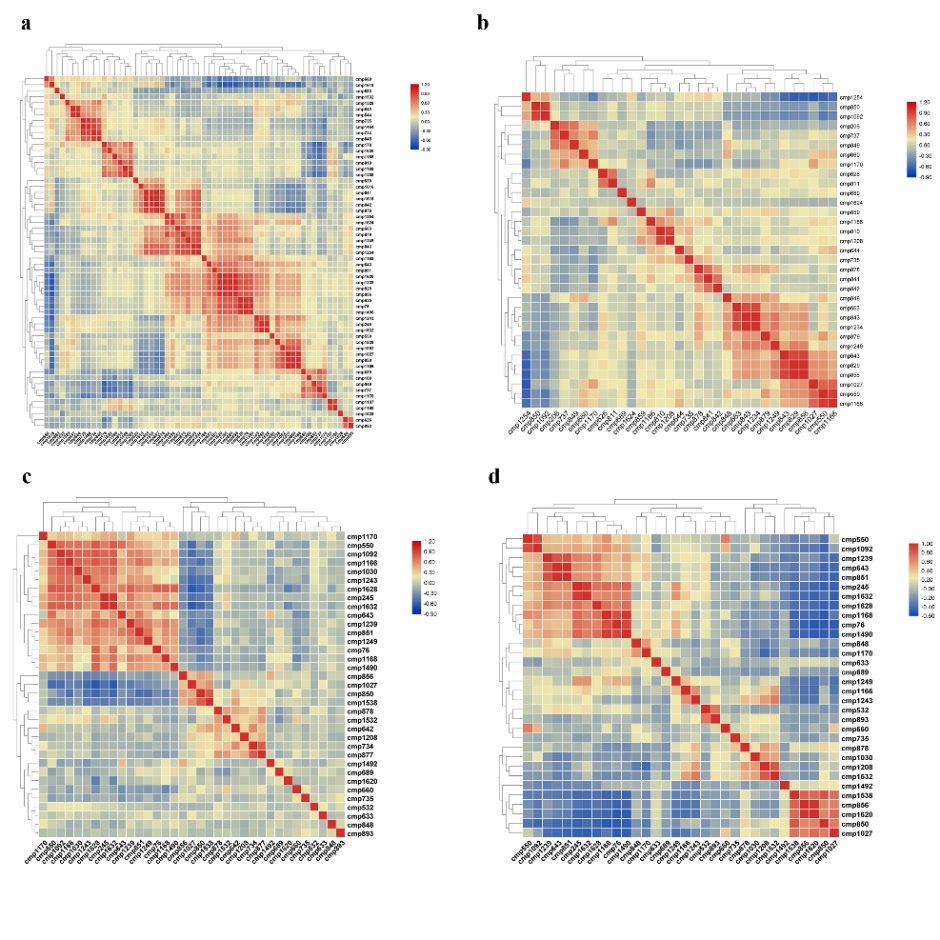
**

**Fig. S3. The Correlation of Flavonoids Detected in Four Tissues in F_1_ Population.**

**a-d** represent young leaves, old leaves, mature pericarp and mature pulp respectively.

**
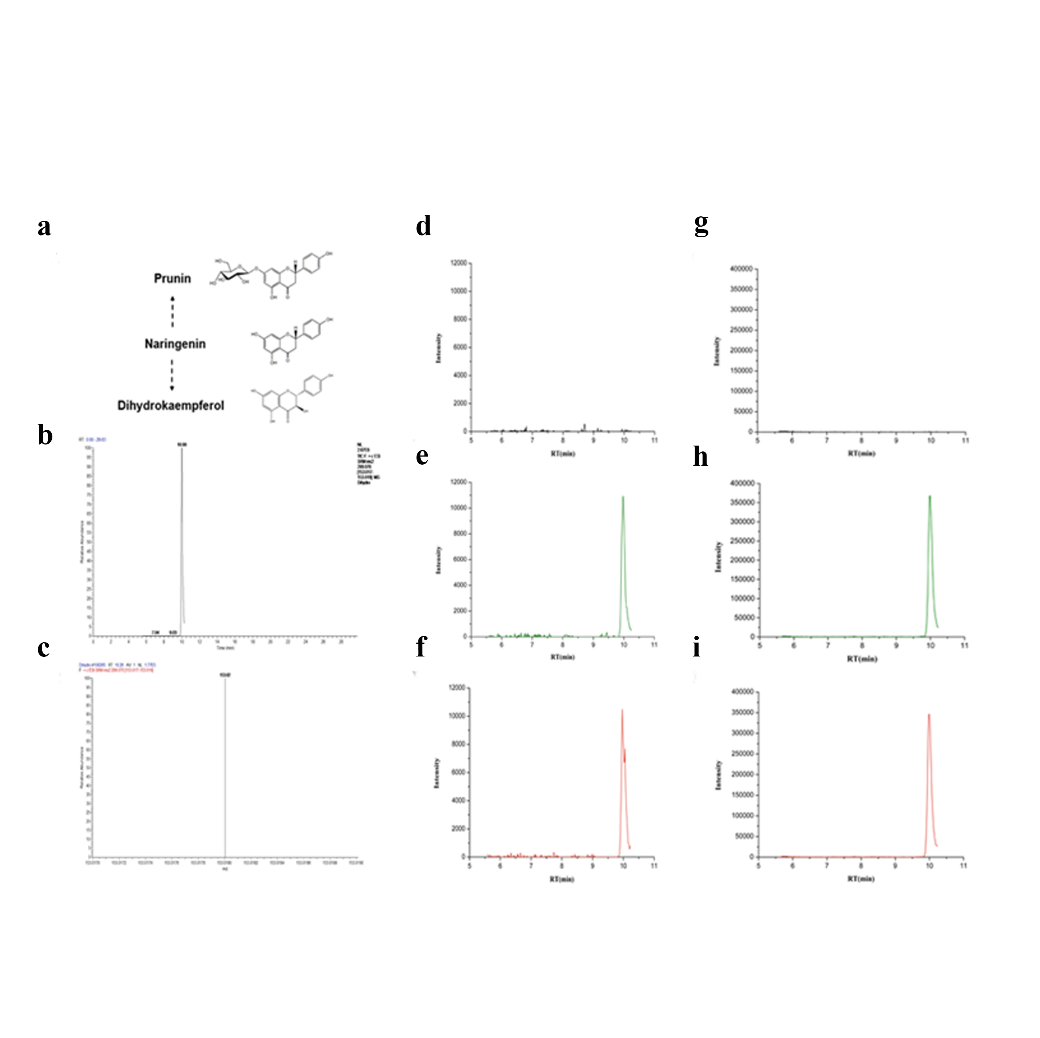
**

**Fig. S4.** **Validation of Candidate Gene *F3H* in Yeast.**

The synthesis pathway of dihydrokaempferol is shown in **a, b** and **c** are TIC and bar diagram of dihydrokaempferol standard, respectively. The retention time of standard is at 10 minutes. The TIC of dihydrokaempferol of precipitation part in **d** (empty PESC-URA lines), **e** (*C. reticulata* overexpression line), **f** (*P. trifoliata* orange overexpression line) and the TIC of dihydrokaempferol of supernatant part in **g** (empty PESC-URA lines), **h** (*C. reticulata* overexpression line), **i** (*P. trifoliata* orange overexpression line) are shown.


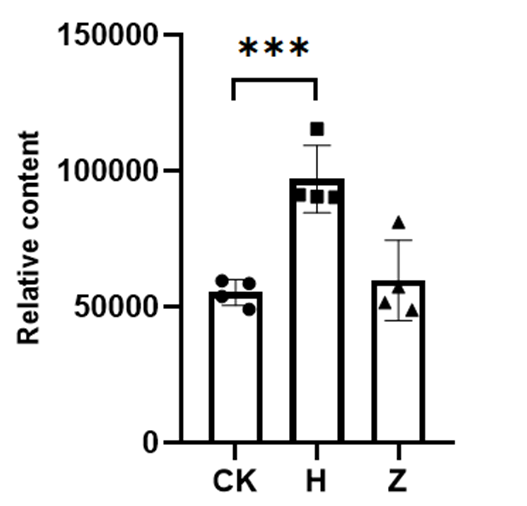


**Fig. S5. The Relative Content of Dihydrokaempferol in Leaves of Transient *N. benthamiana* with Naringenin Added.**

CK represents the empty vector line, H represents *C. reticulata* overexpression line and Z represents *P. trifoliata* overexpression line. n=4. (“***” means *p*<0.001).

**
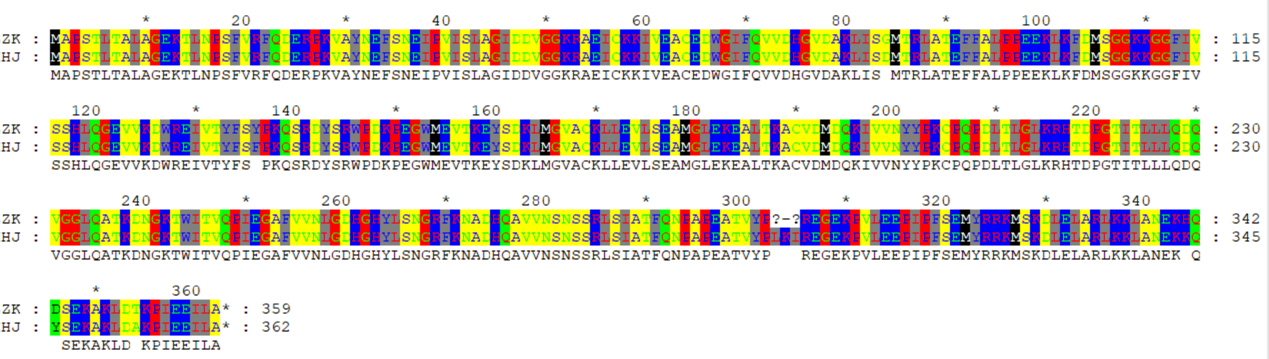
**

**Fig. S6. The Amino Acid Sequence Alignment in Coding Region of *F3H* Between *C. reticulata* and *P. trifoliata*.**

ZK and HJ represent the amino acid sequence of *F3H* in *P. trifoliata* and *C. reticulata*, respectively.

**
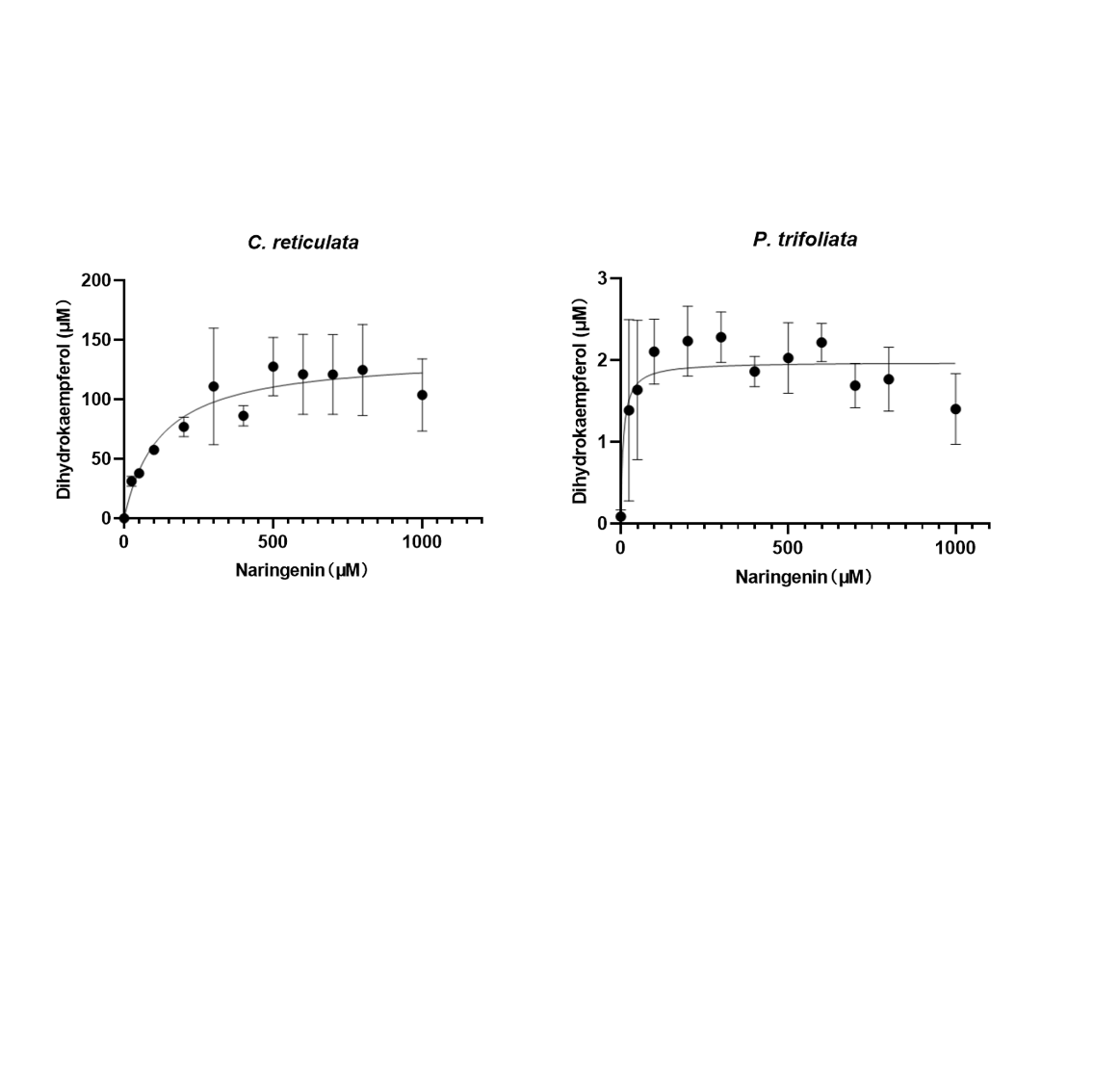
**

**Fig. S7. The Michaelis-Menten Equations of F3H Recombinant Proteins with *C. reticulata* and *P. trifoliata* Alleles.**

**
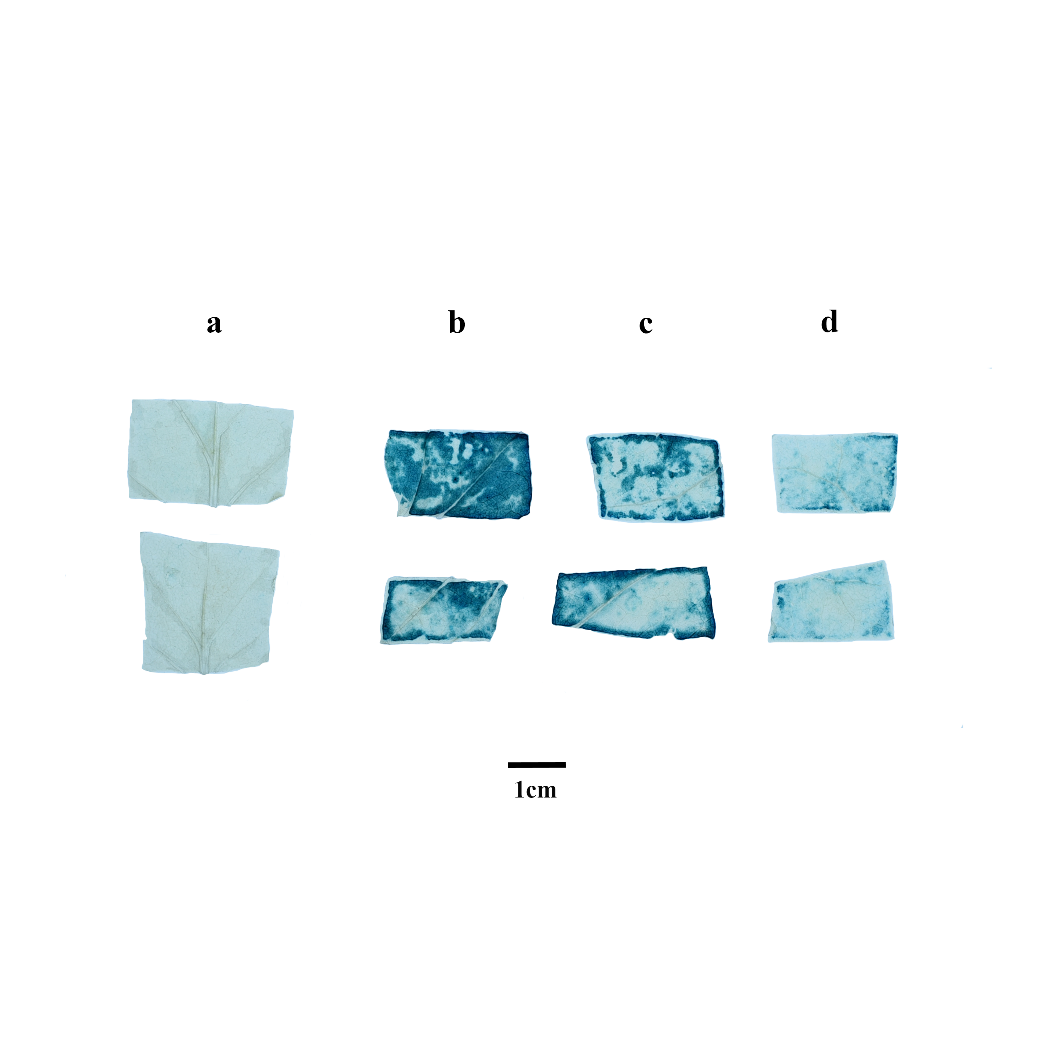
**

**Fig. S8. The Gus Staining of Transient Tobacco.**

**a** represents the result of negative control, **b** represents the result of *P. trifoliata* promoter allele A (short deletion), **c** represents the result of *P. trifoliata* promoter allele B (long deletion) and **d** represents the result of *C. reticulata* promoter, respectively.

**
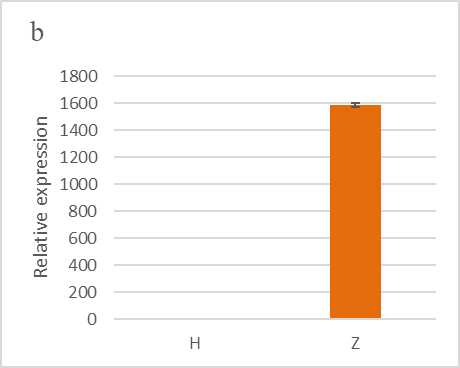

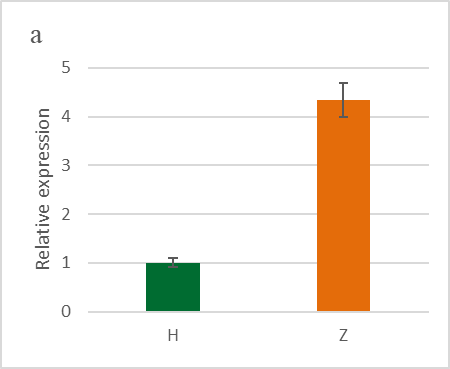
**

**Fig. S9. The Relative Expression of *F3H* in Two Tissues between *C. reticulata* and *P. trifoliata*.**

H represents *C. reticulata and* Z represents *P. trifoliata.* **a** represents relative expression level in young leaf and **b** represents relative expression level in mature pulp by qRT-PCR, n=3.


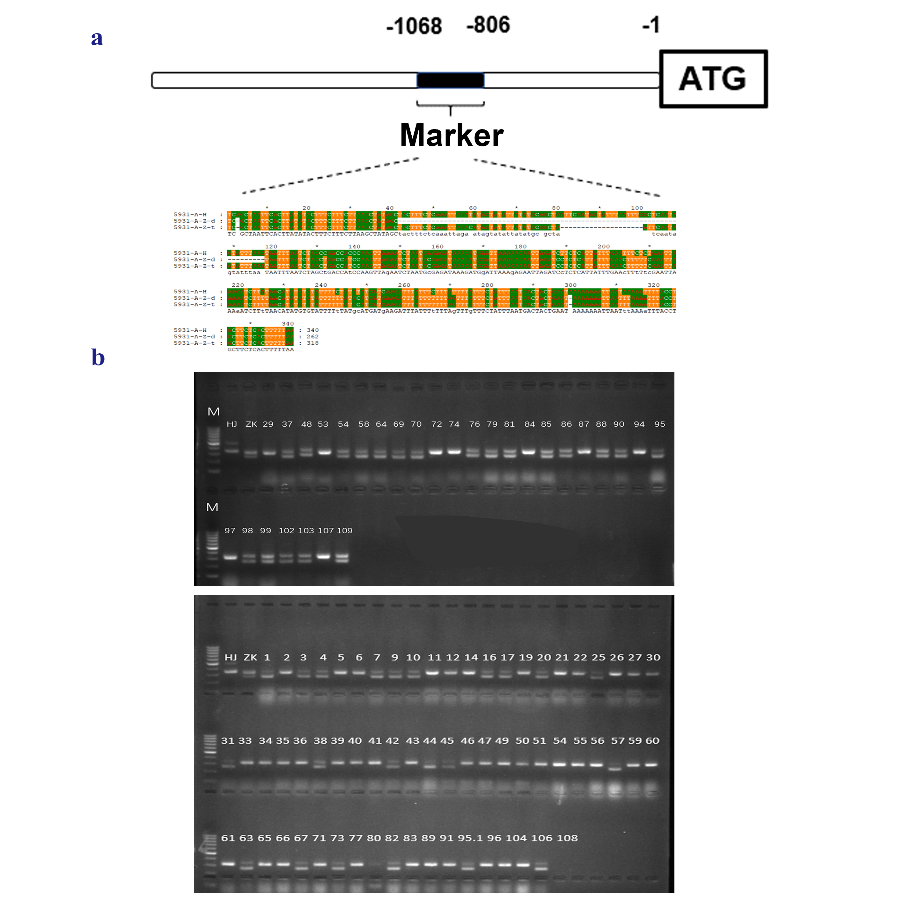


**Fig. S10. The Information of Marker.**

**a** is the sequence alignment, 5931-A-H represents the sequence of Marker in *C. reticulata*, 5931-A-Z-d and 5931-A-Z-t represent the two sequence types of Marker in *P. trifoliata*; **b** is the gel electrophoretogram of two parents and 93 F_1_ siblings. M represents marker(100bp).
