# Supplementary material for: Multiomics-based dissection of citrus flavonoid metabolism using a Citrus reticulata × Poncirus trifoliata population
Source: Hortic Res. 2021 Mar 1;8:56. doi: 10.1038/s41438-021-00472-8 (PMC7917093; doi:10.1038/s41438-021-00472-8)
Supplement: Supplementary file 4 — Data S2 [file 41438_2021_472_MOESM4_ESM.doc]

* 20 * 40 * 60 * 80 * 100
5931-H : GTAGGTATGGATATATGATTTCAGTAAATTAAAAATTATAGAATTAAATAAAATCATCGTCATATTATAAAGTATTTAAATTATTTAAAAAATCTAAAATTTTGAAAC : 108
5931-Z1 : GTAGGTATGGATATATGATTTCAGTAAATTAAAAATTATAGAATTAAATAAAATCATCGTCATATTATAAAGTATTTAAATTATTTAAAAAATCTAAAATTTTGAAAC : 108
5931-Z2 : GTAGGTATGGATATATGATTTCAGTAAATTAAAAATTATAGAATTAAATAAAATCATCGTCATATTATAAAGTATTTAAATTATTTAAAAAATCTAAAATTTTGAAAC : 108
 GTAGGTATGGATATATGATTTCAGTAAATTAAAAATTATAGAATTAAATAAAATCATCGTCATATTATAAAGTATTTAAATTATTTAAAAAATCTAAAATTTTGAAAC

 * 120 * 140 * 160 * 180 * 200 *
5931-H : AATATCTTAAGATGATATCAAAAGTAAAAAATATTTGAAAAGGAGAGAAAAGAAAACATAACAATATTTTAAGGTTGAATTAATAAGAATTATATTGTATGATTTTCT : 216
5931-Z1 : AATATCTTAAGATGATATCAAAAGTAAAAAATATTTGAAAAGGAGAGAAAAGAAAACATAACAATATTTTAAGGTTGAATTAATAAGAATTATATTGTATGATTTTCT : 216
5931-Z2 : AATATCTTAAGATGATATCAAAAGTAAAAAATATTTGAAAAGGAGAGAAAAGAAAACATAACAATATTTTAAGGTTGAATTAATAAGAATTATATTGTATGATTTTCT : 216
 AATATCTTAAGATGATATCAAAAGTAAAAAATATTTGAAAAGGAGAGAAAAGAAAACATAACAATATTTTAAGGTTGAATTAATAAGAATTATATTGTATGATTTTCT

 220 * 240 * 260 * 280 * 300 * 320
5931-H : TTTAGTTATAACTTATTTATATTAGGTAAAAATAAGAAGAAAATTTATTAACCAACAGGATTTATATATAATGAAAATTGAGGTGGGGTGGGAGTGAGGTGGGGAGTA : 324
5931-Z1 : TTTAGTTATAACTTATTTATATTAGGTAAAAATAAGAAGAAAATTTATTAACCAACAGGATTTATATATAATGAAAATTGAGGTGGGGTGGGAGTGAGGTGGGGAGTA : 324
5931-Z2 : TTTAGTTATAACTTATTTATATTAGGTAAAAATAAGAAGAAAATTTATTAACCAACAGGATTTATATATAATGAAAATTGAGGTGGGGTGGGAGTGAGGTGGGGAGTA : 324
 TTTAGTTATAACTTATTTATATTAGGTAAAAATAAGAAGAAAATTTATTAACCAACAGGATTTATATATAATGAAAATTGAGGTGGGGTGGGAGTGAGGTGGGGAGTA

 * 340 * 360 * 380 * 400 * 420 *
5931-H : CAAAACTATATCTCACCTCATAGAAATTATTTGTTCCCCATTATCCGCTCCATCCTCGAAAATTATCTAATTTTTTTTTTCATTTAAGATGGCCCCCATGAGACCCCA : 432
5931-Z1 : CAAAACTATATCTCACCTCATAGAAATTATTTGTTCCCCATTATCCGCTCCATCCTCGAAAATTATCTAATTTTTTTTTTCATTTAAGATGGCCCCCATGAGACCCCA : 432
5931-Z2 : CAAAACTATATCTCACCTCATAGAAATTATTTGTTCCCCATTATCCGCTCCATCCTCGAAAATTATCTAATTTTTTTTTTCATTTAAGATGGCCCCCATGAGACCCCA : 432
 CAAAACTATATCTCACCTCATAGAAATTATTTGTTCCCCATTATCCGCTCCATCCTCGAAAATTATCTAATTTTTTTTTTCATTTAAGATGGCCCCCATGAGACCCCA

 440 * 460 * 480 * 500 * 520 * 540
5931-H : AATCCGTAGAGAAATTTCCATCCCCAGCCAGGCAAGGTAAATGCATGCTTCATGAACGACCAGTCGAGCAGTTAAAGTTAGGTTTAAATCGGGTGCGACCATACCAGC : 540
5931-Z1 : AATCCGTAGAGAAATTTCCATCCCCAGCCAGGCAAGGTAAATGCATGCTTCATGAACGACCAGTCGAGCAGTTAAAGTTAGGTTTAAATCGGGTGCGACCATACCAGC : 540
5931-Z2 : AATCCGTAGAGAAATTTCCATCCCCAGCCAGGCAAGGTAAATGCATGCTTCATGAACGACCAGTCGAGCAGTTAAAGTTAGGTTTAAATCGGGTGCGACCATACCAGC : 540
 AATCCGTAGAGAAATTTCCATCCCCAGCCAGGCAAGGTAAATGCATGCTTCATGAACGACCAGTCGAGCAGTTAAAGTTAGGTTTAAATCGGGTGCGACCATACCAGC

 * 560 * 580 * 600 * 620 * 640
5931-H : ACTAATGTACCGAATCCCATCAGAACTCCGCAGTTAAGCGTGCTTGGACGAGAGCAGTACTAAGATGAGTGACCTCTTGGAAAGTCCCCGTGTTGCACACCTCCTTTT : 648
5931-Z1 : ACTAATGTACCGAATCCCATCAGAACTCCGCAGTTAAGCGTGCTTGGACGAGAGCAGTACTAAGATGAGTGACCTCTTGGAAAGTCCCCGTGTTGCACACCTCCTTTT : 648
5931-Z2 : ACTAATGTACCGAATCCCATCAGAACTCCGCAGTTAAGCGTGCTTGGACGAGAGCAGTACTAAGATGAGTGACCTCTTGGAAAGTCCCCGTGTTGCACACCTCCTTTT : 648
 ACTAATGTACCGAATCCCATCAGAACTCCGCAGTTAAGCGTGCTTGGACGAGAGCAGTACTAAGATGAGTGACCTCTTGGAAAGTCCCCGTGTTGCACACCTCCTTTT

 * 660 * 680 * 700 * 720 * 740 *
5931-H : GAAGTTATATCAAAAAAAAAAAGTTAAGTTTAAATCACGTGATGTGGTCTCAACTATACCTTACATATGACCAAATAAATTATTTATTCTATTCAGCTAATTCACTTA : 756
5931-Z1 : GAAGTTATATCAAAAAAAAAAAGTTAAGTTTAAATCACGTGATGTGGTCTCAACTATACCTTACATATGACCAAATAAATTATTTATTCTATTC-GCTAATTCACTTA : 755
5931-Z2 : GAAGTTATATCAAAAAAAAAAAGTTAAGTTTAAATCACGTGATGTGGTCTCAACTATACCTTACATATGACCAAATAAATTATTTATTCTATTC-GCTAATTCACTTA : 755
 GAAGTTATATCAAAAAAAAAAAGTTAAGTTTAAATCACGTGATGTGGTCTCAACTATACCTTACATATGACCAAATAAATTATTTATTCTATTC GCTAATTCACTTA

 760 * 780 * 800 * 820 * 840 * 860
5931-H : TATACTTTCTTTCTTAAGCTATAGCTACTTTCTCAAATTAGATATAGTATATTATATGCAGCTAATTCAATAGTATTTAATTTGGCTCAATAGTATTTAAGTAATTTA : 864
5931-Z1 : TATACTTTCTTTCTTAAGCTATAGC----------------------------------------------------------------------------TAATTTA : 787
5931-Z2 : TATACTTTCTTTCTTAAGCTATAGCTACTTTCTCAAATTAGACATAGTATATTATATGCGGCTAAT--------------------TCAATAGTATTTAAATAATTTA : 843
 TATACTTTCTTTCTTAAGCTATAGCtactttctcaaattaga atagtatattatatgc gctaat tcaatagtatttaa TAATTTA

 * 880 * 900 * 920 * 940 * 960 *
5931-H : ATCTAGCCGACCACCCAAGTTAAAATCTAATGAGAGATAAAGATGAATTAAAAAGAATTAGATCTTCTCATTATTTGAATTTCTCTGAATTAAAGATCTTATAACATA : 972
5931-Z1 : ATCTAGCTGACCATCCAAGTTAGAATCTAATGCGAGATAAAGATGGATTAAAGAGAATTAGATCCTCTCATTATTTGAACTTTTTCGAATTAAAAATCTTTTAACATA : 895
5931-Z2 : ATCTAGCTGACCATCCAAGTTAGAATCTAATGCGAGATAAAGATGGATTAAAGAGAATTAGATCCTCTCATTATTTGAACTTTTTCGAATTAAAAATCTTTTAACATA : 951
 ATCTAGCtGACCAtCCAAGTTAgAATCTAATGcGAGATAAAGATGgATTAAAgAGAATTAGATCcTCTCATTATTTGAAcTTtTtcGAATTAAAaATCTTtTAACATA

 980 * 1000 * 1020 * 1040 * 1060 * 1080
5931-H : TGTGTATTTTCTATATATGATCAAGATTTATTTCTTTATTTTATTTCTATTTAATGACTACTGAATAAAAAAAATTAATAAAAATTTTACCTGCTTCTCACTTTTTAA : 1080
5931-Z1 : TGTGTATTTTTTATGCATGATGAAGATTTATTTTTTTAGTTTGTTTCTATTTAATGACTACTGAAT-AAAAAAATTAATTTAAAATTTACCTGCTTCTCACTTTTTAA : 1002
5931-Z2 : TGTGTATTTTTTATGCATGATGAAGATTTATTTTTTTAGTTTGTTTCTATTTAATGACTACTGAAT-AAAAAAATTAATTTAAAATTTACCTGCTTCTCACTTTTTAA : 1058
 TGTGTATTTTtTATgcATGATgAAGATTTATTTtTTTAgTTTgTTTCTATTTAATGACTACTGAAT AAAAAAATTAATttAAAaTTTACCTGCTTCTCACTTTTTAA

 * 1100 * 1120 * 1140 * 1160 * 1180
5931-H : AAATGATAATAGGGTAAAAAAAATCCTCCACTTTGTGAAAGGATAACGGGGGAAGGCGTTGGCACATAAACTAAAAAAAAAGGTTGTTCGTATAAAATTGGGATAAAT : 1188
5931-Z1 : AAATGATAATAGGGTAAAAAAAATCCTCCACTTTGTGAAAGGATAACGGGGGAAGGCGTTGGCACATAAACT-AAAAAAAAGGTTGTTCGTATAAAATTGGGATAAAT : 1109
5931-Z2 : AAATGATAATAGGGTAAAAAAAATCCTCCACTTTGTGAAAGGATAACGGGGGAAGGCGTTGGCACATAAACT-AAAAAAAAGGTTGTTCGTATAAAATTGGGATAAAT : 1165
 AAATGATAATAGGGTAAAAAAAATCCTCCACTTTGTGAAAGGATAACGGGGGAAGGCGTTGGCACATAAACT AAAAAAAAGGTTGTTCGTATAAAATTGGGATAAAT

 * 1200 * 1220 * 1240 * 1260 * 1280 *
5931-H : AAAAATATGTGGTGACCGAATTTACATGGCATTTGACTTGACAGCCAATCCGTACTTTGCATGCCGAAGACGAATACGGCGCCACGTAGCTAGGTTGCCAAGTTTGTT : 1296
5931-Z1 : AAAAATATGTGGTGACCGAATTTACATGGCATTTGACTTGACAGCCAATCCGTACTTTGCATGCCGAAGACGAATACGGCGCCACGTAGCTAGGTTGCCAAGTTTGTT : 1217
5931-Z2 : AAAAATATGTGGTGACCGAATTTACATGGCATTTGACTTGACAGCCAATCCGTACTTTGCATGCCGAAGACGAATACGGCGCCACGTAGCTAGGTTGCCAAGTTTGTT : 1273
 AAAAATATGTGGTGACCGAATTTACATGGCATTTGACTTGACAGCCAATCCGTACTTTGCATGCCGAAGACGAATACGGCGCCACGTAGCTAGGTTGCCAAGTTTGTT

 1300 * 1320 * 1340 * 1360 * 1380 * 1400
5931-H : GGAATTCGTTTATCTGATTTTTATATGTATAACAAAATAATAAAAATATTTATTTCTGAATTTCTGTATACTAATAAAAATTATGCAGTTAAAACTTAAACGAACGCA : 1404
5931-Z1 : GGAATTCGTTTATCTGATTTTTATATGTATAACAAAATAATAAAAATATTTATTTCTGAATTTCTGTATACTAATAAAAATTATGCAGTTAAAACTTAAACGAACGCA : 1325
5931-Z2 : GGAATTCGTTTATCTGATTTTTATATGTATAACAAAATAATAAAAATATTTATTTCTGAATTTCTGTATACTAATAAAAATTATGCAGTTAAAACTTAAACGAACGCA : 1381
 GGAATTCGTTTATCTGATTTTTATATGTATAACAAAATAATAAAAATATTTATTTCTGAATTTCTGTATACTAATAAAAATTATGCAGTTAAAACTTAAACGAACGCA

 * 1420 * 1440 * 1460 * 1480 * 1500 *
5931-H : CTGGTCAATAGAGAGTGATCCATTCAATTCAATGCTTAAAAAAAGTTAAAACAATTGAGATAAGATATTCTATATAAAGCTCCTCCGTTACCCAACCACGTAGACTCT : 1512
5931-Z1 : CTGGTCAATAGAGAGTGATCCATTCAATTCAATGCTTAAAAAAAGTTAAAACAATTGAGATAAGATATTCTATATAAAGCTCCTCCGTTACCCAACCACGTAGACTCT : 1433
5931-Z2 : CTGGTCAATAGAGAGTGATCCATTCAATTCAATGCTTAAAAAAAGTTAAAACAATTGAGATAAGATATTCTATATAAAGCTCCTCCGTTACCCAACCACGTAGACTCT : 1489
 CTGGTCAATAGAGAGTGATCCATTCAATTCAATGCTTAAAAAAAGTTAAAACAATTGAGATAAGATATTCTATATAAAGCTCCTCCGTTACCCAACCACGTAGACTCT

 1520 * 1540 * 1560 * 1580 * 1600 * 1620
5931-H : AGAAGCACGTATGCTGATACGCATGTAGGGTGGTAGATGGATGCCACTTGAAGTGTTAAAACATTTAAGAGGATGGTAGTTCACGTGTTAAGAGGTAGCCACAGCTGC : 1620
5931-Z1 : AGAAGCACGTATGCTGATACGCATGTAGGGTGGTAGATGGATGCCACTTGAAGTGTTAAAACATTTAAGAGGATGGTAGTTCACGTGTTAAGAGGTAGCCACAGCTGC : 1541
5931-Z2 : AGAAGCACGTATGCTGATACGCATGTAGGGTGGTAGATGGATGCCACTTGAAGTGTTAAAACATTTAAGAGGATGGTAGTTCACGTGTTAAGAGGTAGCCACAGCTGC : 1597
 AGAAGCACGTATGCTGATACGCATGTAGGGTGGTAGATGGATGCCACTTGAAGTGTTAAAACATTTAAGAGGATGGTAGTTCACGTGTTAAGAGGTAGCCACAGCTGC

 * 1640 * 1660 * 1680 * 1700 * 1720
5931-H : CACGCTTTCCAACCGTCTAACATCCGAAGCCGCGTCCCCCTCGTACTCCATATAATATATATACATAGGTGTAAAACATGCACACATGCACATTAACCCCCACAACAC : 1728
5931-Z1 : CACGCTTTCCAACCGTCTAACATCCGAAGCCGCGTCCCCCTCGTACTCCATATAATATATATACATAGGTGTAAAACATGCACACATGCACATTAACCCCCACAACAC : 1649
5931-Z2 : CACGCTTTCCAACCGTCTAACATCCGAAGCCGCGTCCCCCTCGTACTCCATATAATATATATACATAGGTGTAAAACATGCACACATGCACATTAACCCCCACAACAC : 1705
 CACGCTTTCCAACCGTCTAACATCCGAAGCCGCGTCCCCCTCGTACTCCATATAATATATATACATAGGTGTAAAACATGCACACATGCACATTAACCCCCACAACAC

 * 1740 * 1760 * 1780 * 1800 * 1820 *
5931-H : AAGCGGCAGTTGCGCATCACCCTTGGACTCACTCTTTTGCTTTTAAACAGATACTTATACATTTGCTCTAATATATTATTATTATTTGTGCTTCATCGATCATCAATT : 1836
5931-Z1 : AAGCGGCAGTTGCGCATCACCCTTGGACTCACTCTTTTGCTTTTAAACAGATACTTATACATTTGCTCTAATATATTATTATTATTTGTGCTTCATCGATCATCAATT : 1757
5931-Z2 : AAGCGGCAGTTGCGCATCACCCTTGGACTCACTCTTTTGCTTTTAAACAGATACTTATACATTTGCTCTAATATATTATTATTATTTGTGCTTCATCGATCATCAATT : 1813
 AAGCGGCAGTTGCGCATCACCCTTGGACTCACTCTTTTGCTTTTAAACAGATACTTATACATTTGCTCTAATATATTATTATTATTTGTGCTTCATCGATCATCAATT


5931-H : C : 1837
5931-Z1 : C : 1758
5931-Z2 : C : 1814
 C
