# Supplementary material for: Multiomics-based dissection of citrus flavonoid metabolism using a Citrus reticulata × Poncirus trifoliata population
Source: Hortic Res. 2021 Mar 1;8:56. doi: 10.1038/s41438-021-00472-8 (PMC7917093; doi:10.1038/s41438-021-00472-8)
Supplement: Supplementary file 3 [file 41438_2021_472_MOESM3_ESM.doc]

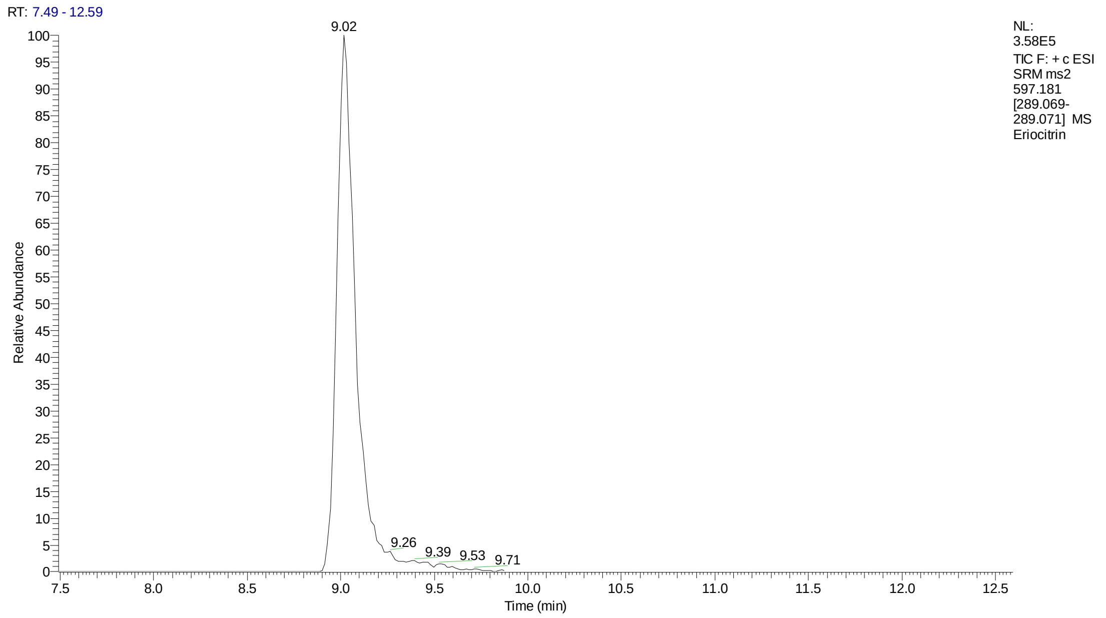
RT: 7.49 - 12.59
NL:
3.58E5
TIC F: + c ESI
SRM ms2
597.181
[289.069-
289.071] MS
Eriocitrin
9.02
9.26
9.39
9.53
9.71
Relative Abundance
Time (min)

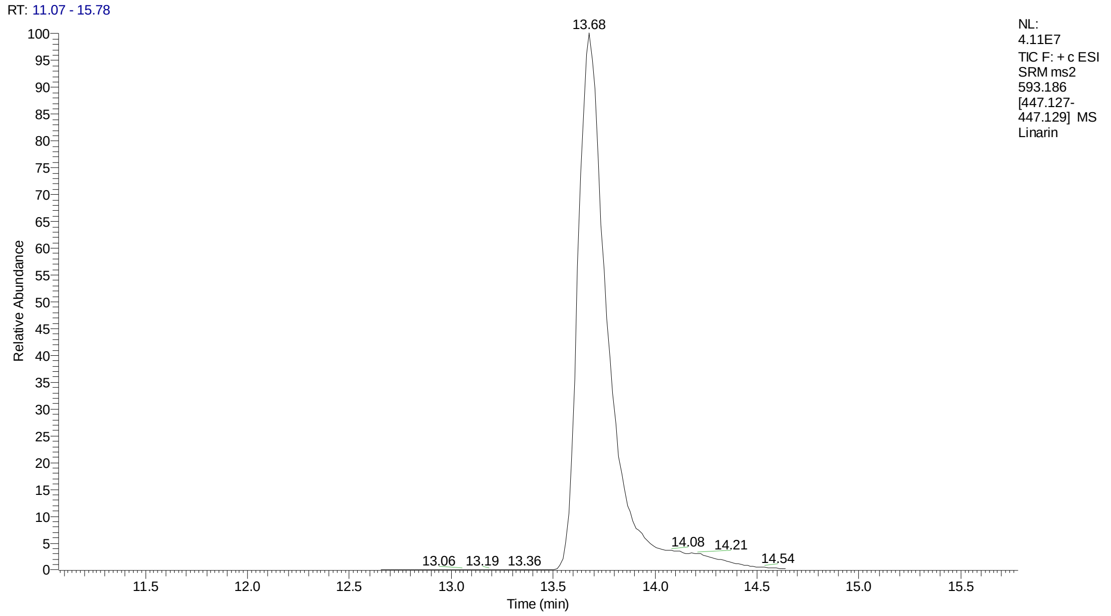
RT: 11.07 - 15.78
NL:
4.11E7
TIC F: + c ESI
SRM ms2
593.186
[447.127-
447.129] MS
Linarin
13.68
13.06
13.19
13.36
14.08
14.21
14.54
Relative Abundance
Time (min)

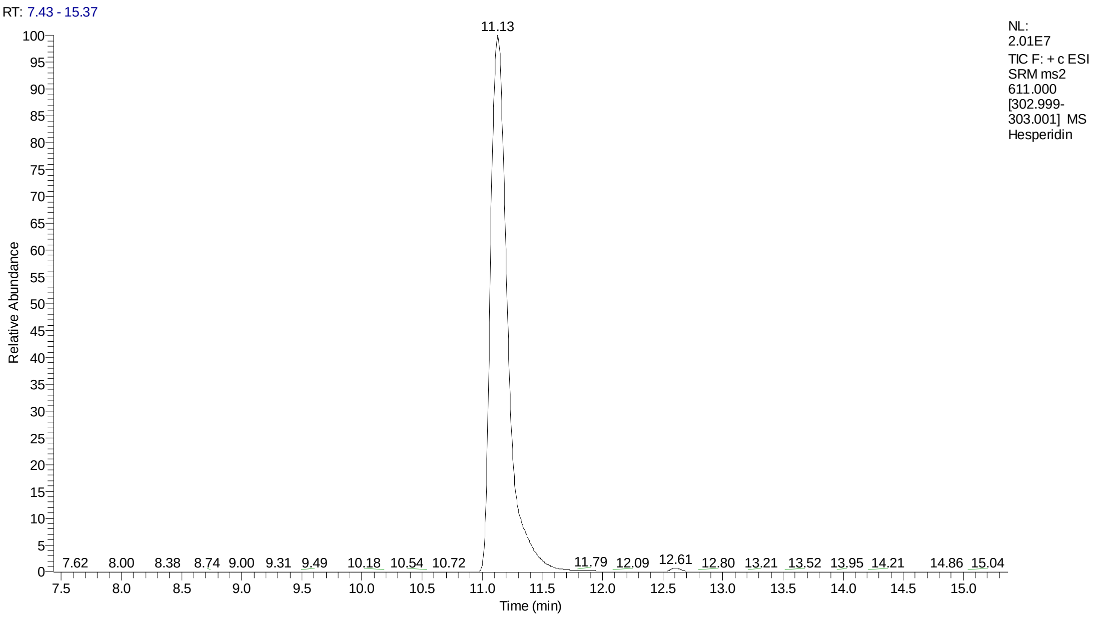

RT: 7.43 - 15.37
11.13
NL:
2.01E7
TIC F: + c ESI
SRM ms2
611.000
[302.999-
303.001] MS
Hesperidin
Relative Abundance
Time (min)
7.62 8.00 8.38 8.74 9.00 9.31 9.49 10.18 10.54 10.72 11.79 12.09 12.61 12.80 13.21 13.52 13.95 14.21 14.86 15.04

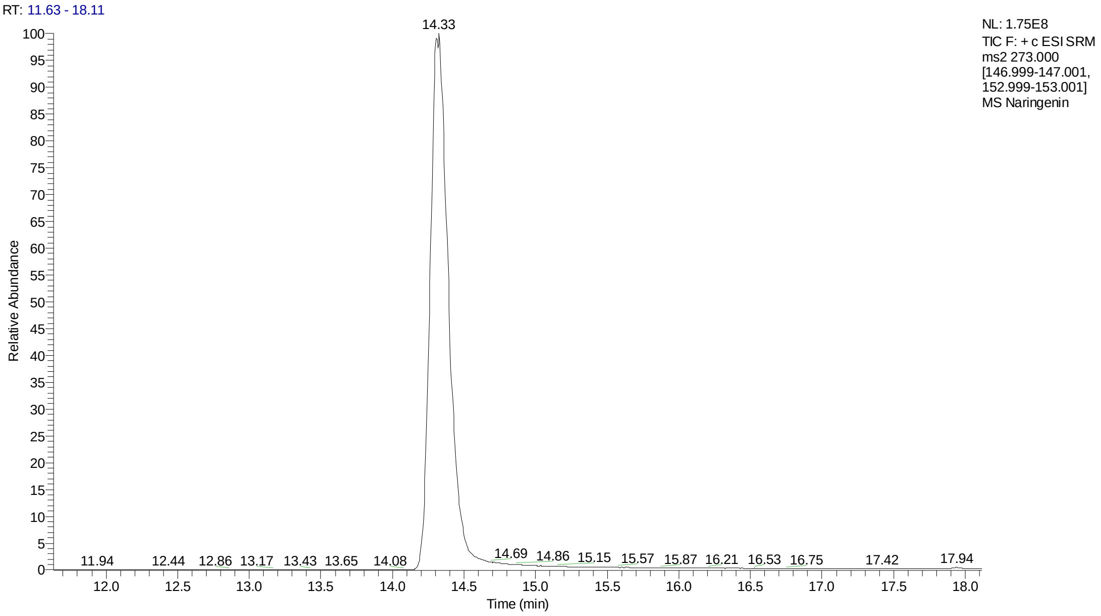

RT: 11.63 - 18.11
14.33
NL: 1.75E8
TIC F: + c ESI SRM
ms2 273.000
[146.999-147.001,
152.999-153.001]
MS Naringenin
Relative Abundance
Time (min)
11.94 12.44 12.86 13.17 13.43 13.65 14.08 14.69 14.86 15.15 15.57 15.87 16.21 16.53 16.75 17.42 17.94

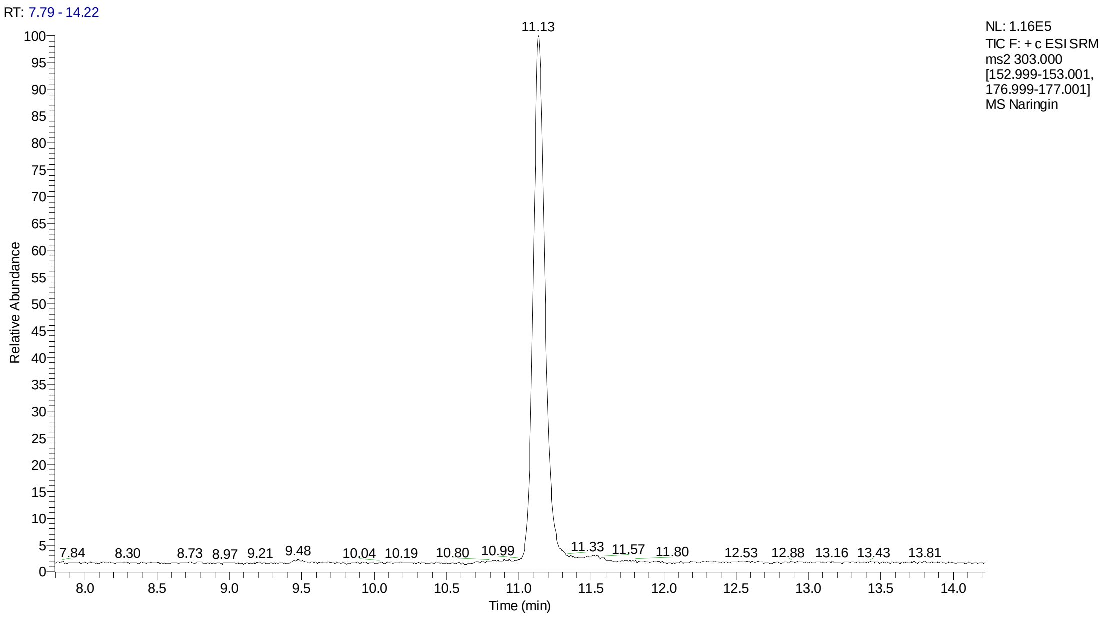
RT: 7.79 - 14.22
NL: 1.16E5
TIC F: + c ESI SRM ms2 303.000 [152.999-153.001, 176.999-177.001] MS Naringin
Relative Abundance
Time (min)
11.13
7.84
8.30
8.73
8.97
9.21
9.48
10.04
10.19
10.80
10.99
11.33
11.57
11.80
12.53
12.88
13.16
13.43
13.81

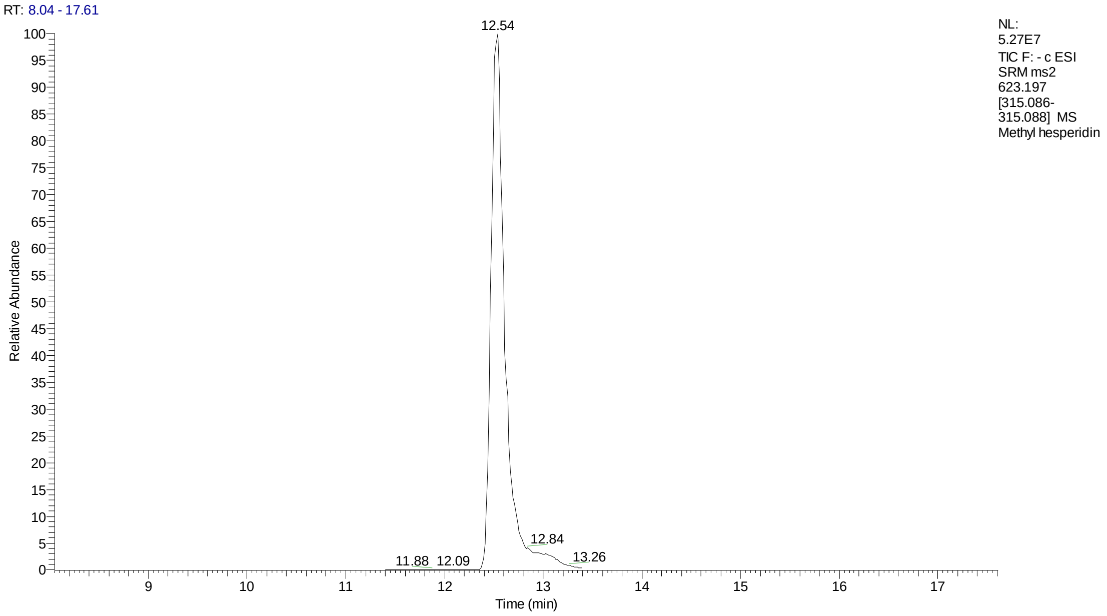
RT: 8.04 - 17.61
NL: 5.27E7
TIC F: - c ESI SRM ms2 623.197 [315.086-315.088] MS Methyl hesperidin
Relative Abundance
Time (min)
12.54
11.88
12.09
12.84
13.26

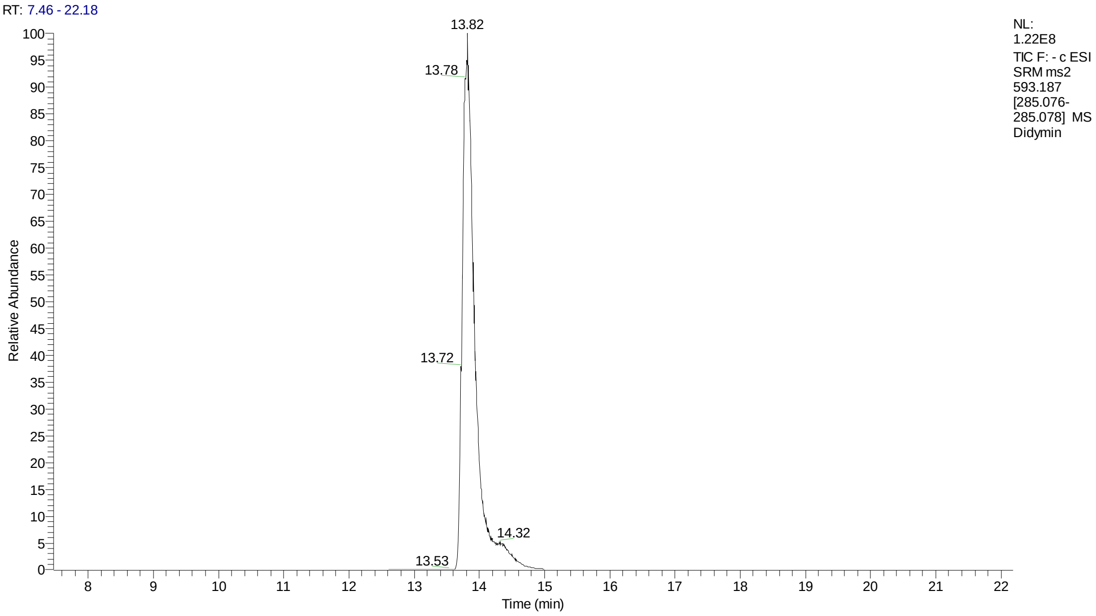
RT: 7.46 - 22.18
NL:
1.22E8
TIC F: - c ESI
SRM ms2
593.187
[285.076-
285.078] MS
Didymin
13.82
13.78
13.72
14.32
13.53
Relative Abundance
Time (min)

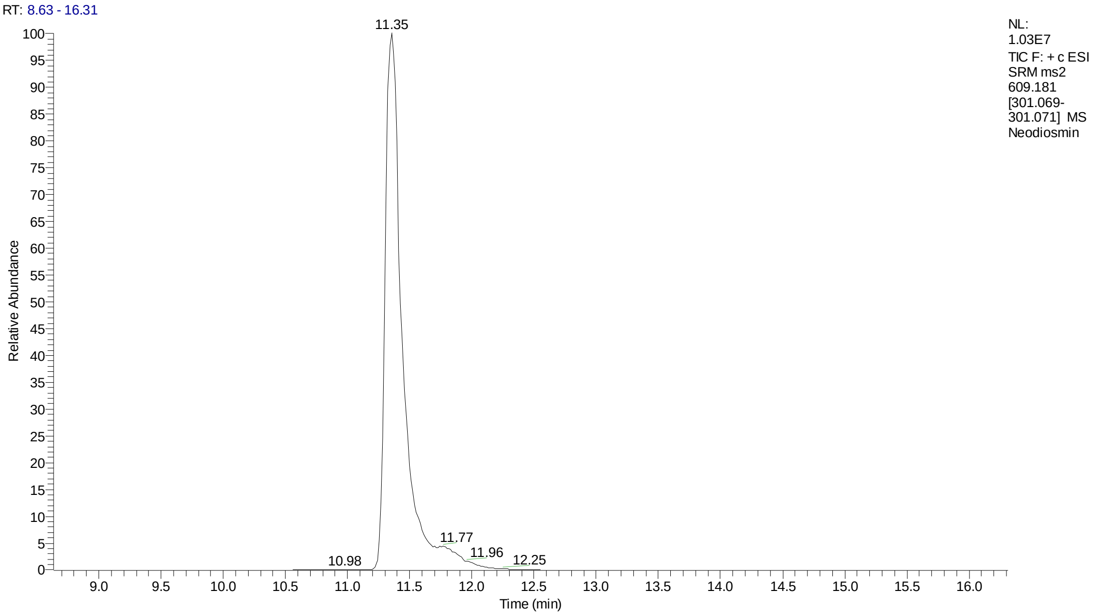
RT: 8.63 - 16.31
NL:
1.03E7
TIC F: + c ESI
SRM ms2
609.181
[301.069-
301.071] MS
Neodiosmin
11.35
11.77
11.96
12.25
10.98
Relative Abundance
Time (min)

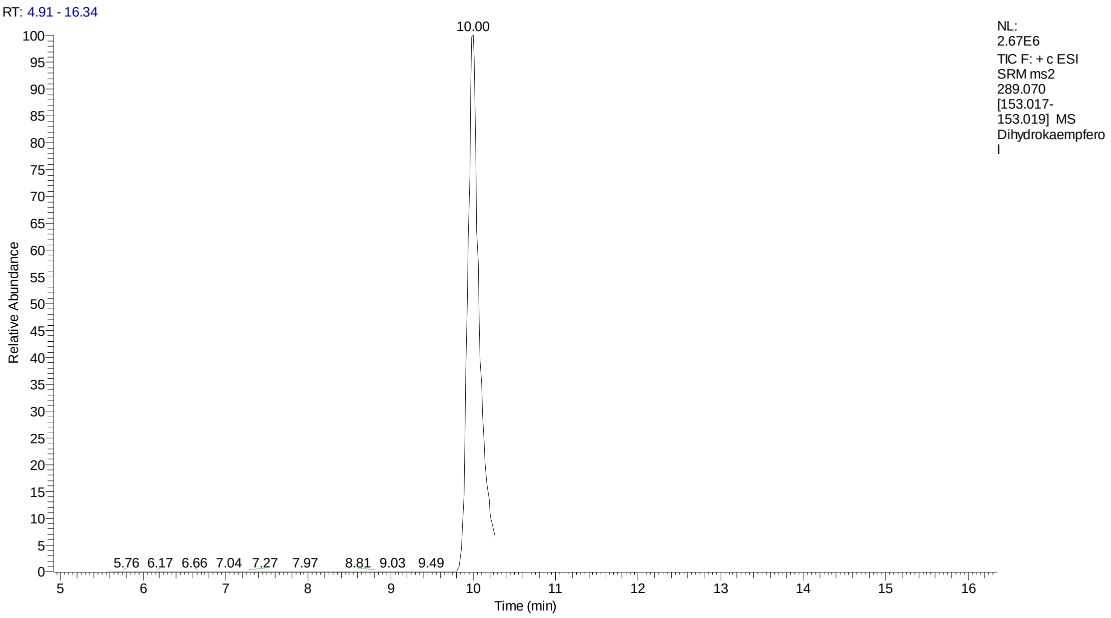
RT: 4.91 - 16.34
NL:
2.67E6
TIC F: + c ESI
SRM ms2
289.070
[153.017-
153.019] MS
Dihydrokaempfero
l
Relative Abundance
Time (min)
10.00
5.76 6.17 6.66 7.04 7.27 7.97 8.81 9.03 9.49

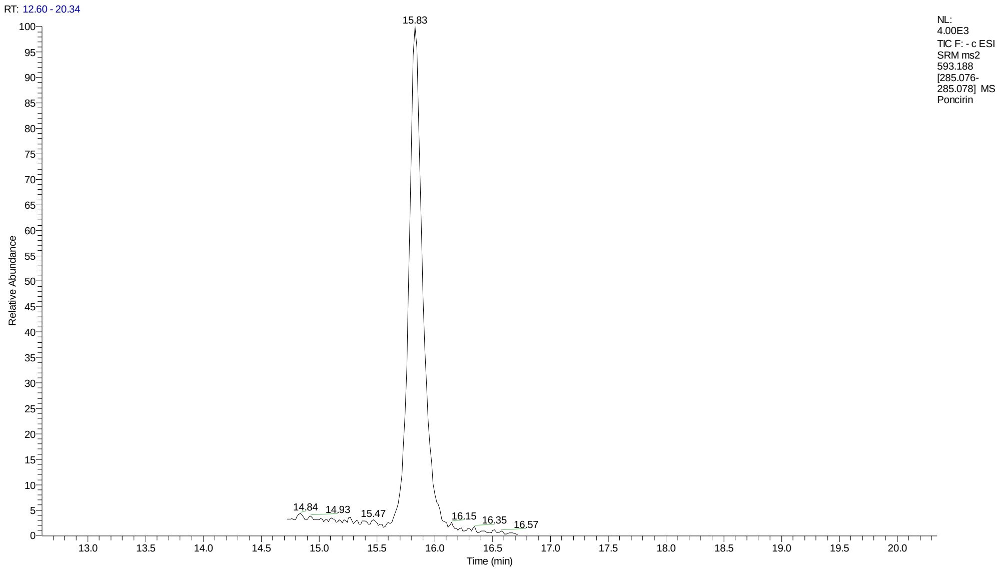
RT: 12.60 - 20.34
NL:
4.00E3
TIC F: - c ESI
SRM ms2
593.188
[285.076-
285.078] MS
Poncirin
Relative Abundance
Time (min)
15.83
14.84 14.93 15.47 16.15 16.35 16.57
