# Supplementary material for: Multiomics-based dissection of citrus flavonoid metabolism using a Citrus reticulata × Poncirus trifoliata population
Source: Hortic Res. 2021 Mar 1;8:56. doi: 10.1038/s41438-021-00472-8 (PMC7917093; doi:10.1038/s41438-021-00472-8)
Supplement: Supplementary file 2 — Cover letter [file 41438_2021_472_MOESM2_ESM.doc]

Dear Editor:

I would like to submit our manuscript entitled “Multi-omics based dissection of citrus flavonoid metabolism using a *Citrus reticulata* x *Poncirus trifoliata* population” for your consideration of publication in the ‘Horticulture Research’ as a research article.

Flavonoids confer to flavor, nutrition and medicinal value of citrus which is one of the most important fruit crops in the world. Our study applied widely targeted metabolomic and QTL analysis in multiple tissues of *C. reticulata* x *P. trifoliata* F1 segregating population, combining of transcriptomic analysis from diverse citrus varieties. Our study proposed a citrus flavonoid metabolic network covering 80 putative flavonoids and 21 candidate genes, among them the function of *CitF3H* and genetic variants in both the coding and promotor region of *CitF3H* resulted in the variation of dihydrokaempferol content in this population were verified. These results lay a foundation for unraveling citrus flavonoid pathway and provide facilitating resources to support breeding and engineering efforts for citrus quality improvement.

We would like to recommend several experts and specialists in the field of plant metabolites and biology as following: Dr. Eugenio Butelli from John Innes Centre (eugenio.butelli@jic.ac.uk); Dr. Takayuki Tohge from Nara Institute of Science and Technology (tohge@bs.naist.jp); Dr Ric de Vos from Wageningen Plant Research, Wageningen University and Research (ric.devos@wur.nl).

This manuscript is our original work and it has not been submitted to any other journal. We deeply appreciate your consideration of our manuscript, and we look forward to receiving feedbacks from you and the reviewers. If you need more information, please don’t hesitate to contact us at the address below.

Best wishes,

Xiuxin Deng (PhD/Professor) and Weiwei Wen (PhD/Professor)

Key Laboratory of Horticultural Plant Biology (MOE)

Huazhong Agricultural University, Wuhan 430070, China

Tel: + 86-27-87286906

Fax: + 86-27-87281850

Email: xxdeng@mail.hzau.edu.cn; wwwen@mail.hzau.edu.cn
